# Boron Lewis Acid Extraction of Wood Generates High Quality Lignin

**DOI:** 10.1021/acssuschemeng.4c06206

**Published:** 2024-11-14

**Authors:** Theodora E. Leventis, Patrick Judge, Jialiang Zhang, M. Zain H. Kazmi, Marcus B. Foston, Florence J. Williams

**Affiliations:** † The University of Iowa, Iowa City, Iowa 52242-1002, United States; ‡ Washington University in St. Louis, St. Louis, Missouri 63130-4899, United States; § University of Alberta, Edmonton, Alberta T6G 2R3, Canada

**Keywords:** lignin, lignocellulose, lignin condensation, lignin monomers, depolymerization, boron Lewis acids, biopolymers, organic polymers

## Abstract

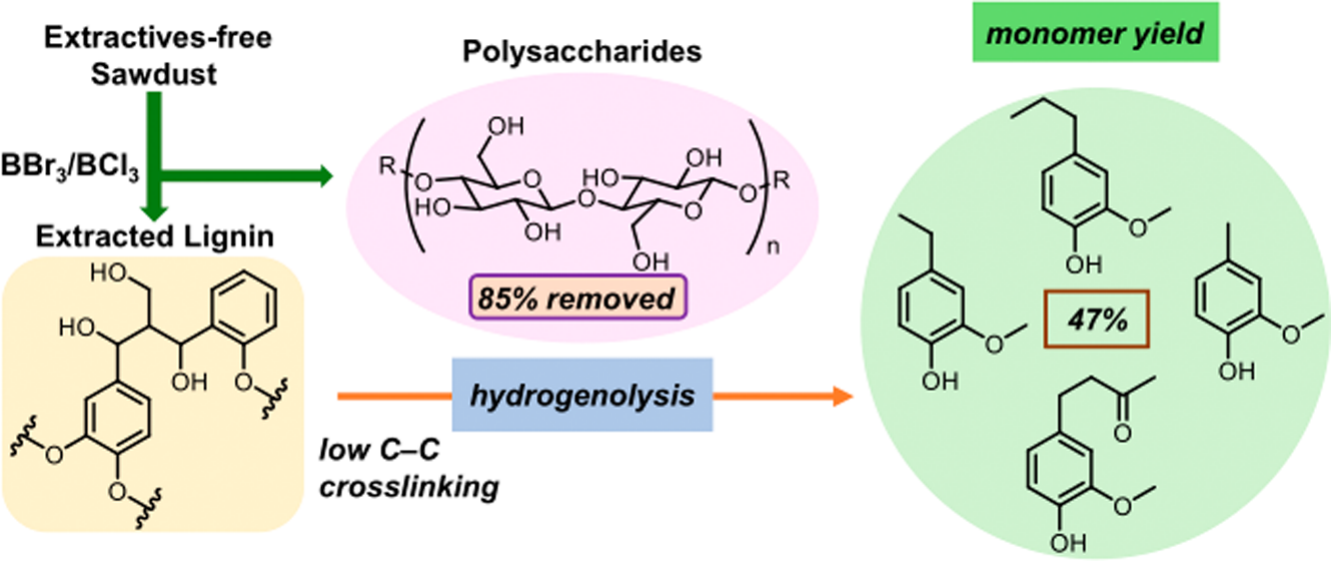

The separation of lignocellulose into lignin, cellulose, and hemicellulose without significantly altering the chemical structures of these component biopolymers remains a modern chemical challenge. Lignin, in particular, has potential as a highly valuable feedstock material but remains underutilized due to the difficulty of generating lignin with low modification and condensation. This work investigates the lignin-rich solids (“boron lignin”) generated from a previously reported boron Lewis acid-mediated lignocellulose separation and concludes that (1) boron Lewis acid extraction removes 80–85% of carbohydrates from the original lignocellulose sample, and (2) the resulting lignin possesses a low condensation level and high similarity to native lignin structure. Residual carbohydrate assessment, depolymerization efficiency analyses, heteronuclear single quantum coherence (HSQC) and solid-state nuclear magnetic resonance (NMR) analyses are discussed, including benchmarking results with alternate lignin sources known to possess low and high condensation levels. Further, two different wood sources (white pine, a softwood, and beechwood, a hardwood) were employed to generate lignin samples. Depolymerization of a white pine-derived boron-lignin produced 47% (±9.5) of extractable monomers, which compares well to a state-of-the-art method to generate low condensed lignin (56 ± 7.8%). An unexpected instability of the oil sample was observed following hydrogenolysis of boron lignin generated from beechwood. Dramatic color changes coupled with precipitation and lowered monomer yields were observed when samples were aged (11% decrease) or concentrated (30% decrease). Based on NMR spectroscopic analyses, this instability is postulated to arise due to boron-mediated demethylation of methoxy sites on the lignin scaffold.

## Abstract 2

Lignin generated using a boron Lewis acid extraction is investigated and characterized, demonstrating high levels of carbohydrate removal while retaining structural similarity to native lignin.

## Introduction

Lignocellulose is the predominant structural material found in all plant cell walls, particularly in woody and fibrous plant tissues.^1^ As a result, the three components of lignocellulose—lignin, cellulose, and hemicellulose—are the most abundant biopolymers on the planet. Cellulose, having a ∼4-billion-dollar commodity market value, is regularly converted into biofuels, chemicals, and other valuable products.^2^ The extraction of cellulose typically involves Brønsted acids and oxidants, resulting in lignin polymer cross-linking and degradation.^3^

Lignin is a nonrepeating polyphenolic polymer connected through ether bonds and possesses a variety of monomers such as coniferyl, sinapyl, and *p*-coumaryl alcohol. Unfortunately, lignin is highly resistant to deconstruction.^4^ As a result, lignin is primarily used for generating heat and power through burning.^5^ However, there is much interest in lignin valorization in the context of sustainable and environmentally friendly biomass utilization. Current industrial scale production of phenolics utilize the cumene process with aromatics obtained from catalytic reformation of fossil-based feedstocks.^6,7^ Overall, this process is energy intensive and inefficient due to the catalytic reformation step (>600 °C, 1–10% yield range).^6^ An attractive alternative would be the depolymerization of lignin to provide useful phenolic monomers in higher overall yield and from abundant and renewable plant sources.^8^ Ideally, such a protocol would also preserve the cellulose fraction to allow for commercial utilization.

Additionally, there has been immense growth in the utilization of lignin for sustainable materials production, including polyurethane alternatives, construction materials, hydrogels, and more.^4,9−11^ The generation of lignin with limited chemical alteration is preferable for these applications because it provides the widest range of chemical utility–subsequent oxidation, condensation, or other derivatization can be performed as desired.

Our group has previously reported a procedure using a 1:1 mixture of boron tribromide (BBr_3_) and boron trichloride (BCl_3_) to selectively separate polysaccharides and lignin without subjecting the lignin sample to high temperatures, oxidants, or concentrated Brønsted acids.^12^ There is reason to suspect that these alternative conditions may be effective in preventing condensation and cross-linking. For instance, avoiding oxidative conditions mitigates oxidative coupling through the intermediacy of quinones or radicals, which have been observed in prior preparations.^13,14^ Additionally, strong Brønsted acids facilitate condensation of the lignin framework, and calculations from Sturgeon and co-workers have demonstrated that the activation energy for this process in a dimeric model lignin is quite low (∼1–11 kcal/mol, depending on lignin features).^15^ These insights have led to speculation that Lewis acids may avoid or lessen these degradation reactions and provide an opportunity to generate lignin with increased retention of native structure.

A number of recent studies have begun to explore Lewis acid reagents in the search for higher quality lignin in lignocellulose separations.^16−26^ Despite several studies correlating increased hardness of the Lewis acid to increased lignin yield,^18^ boron Lewis acids are relatively underexplored in this context. Zhang and co-workers have investigated the use of BBr_3_ to depolymerize lignin samples, but the carbohydrate fraction had been previously removed in these samples by other methods.^27^

There is ample potential for sustainable lignocellulose valorization using boron Lewis acid strategies. Boron trihalide compounds, and related lignin and cellulose reaction products of boron trihalide treatment, are rapidly hydrolyzed with water to generate boric acid and sodium or potassium salts, both of which are considered nontoxic to mammals. Further, our lab’s BBr_3_/BCl_3_-mediated separation is performed at room temperature. If low-condensed lignin is generated, subsequent depolymerization of lignin (which can be done at or below 250 °C, vide infra) compares favorably in terms of energy consumption to the previously mentioned catalytic reformation and cumene process sequence.^6−8^ A current drawback is that boron trihalides are not easily regenerated, and thus far, are needed in stoichiometric amounts. To establish whether further optimization is warranted to address such limitations, the products of lignocellulose separation by BBr_3_/BCl_3_ must be evaluated for their quality and potential utility.

Although the use of boron Lewis acids for separating lignin, cellulose, and hemicellulose is underexplored, it is worth noting that industrial wood products are frequently treated with boric acid or borax to imbue insecticidal and fungicidal properties that extend lifetime and utility.^28^ While boric acid and analogues like borax are known to form monovalent and divalent coordination complexes with sugars and polysaccharides,^29,30^ mechanistic and kinetic studies have demonstrated that boric acid reactivity with wood components (i.e., cellulose) is slow at room temperature, and that the bulk of the boron mass is incorporated through adsorption.^29,31^

**Figure 1:**
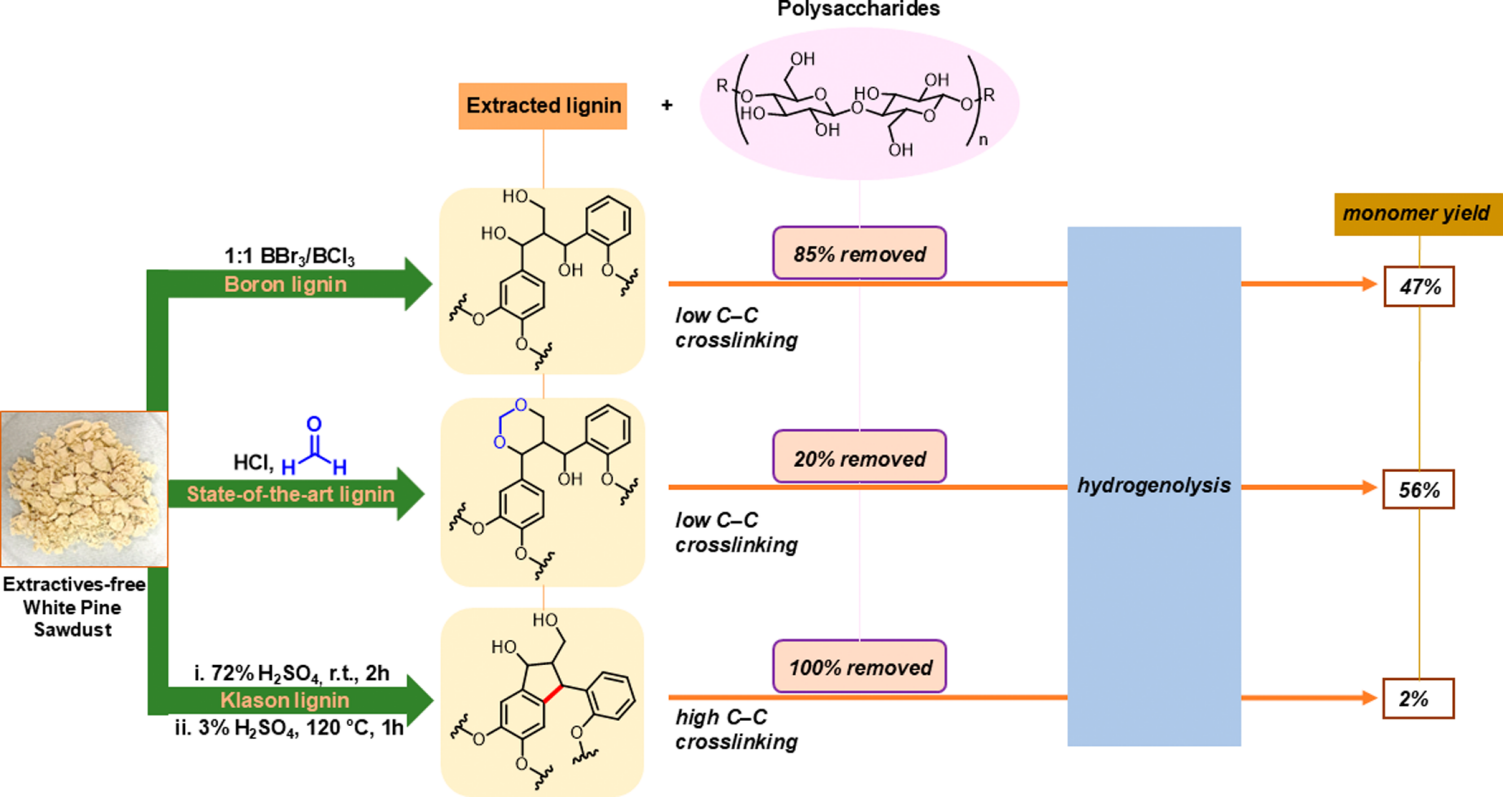
Lignin extraction via three protocols followed by depolymerization to aromatic monomers. Extractives-free white pine sawdust was used to prepare lignin following three different methods: our mixed boron trihalide protocol, the formaldehyde protocol (low-condensed and modified), and the Klason procedure (highly condensed and largely modified).^12,32^ The amount of polysaccharides removed during the separation procedures is noted (wt %), with Klason removing the most sugar (100%) and FA removing the least (20%). The resulting lignin from each method was subjected (separately) to a hydrogenolysis reaction (vide infra). Lignin generated by mixed boron trihalides provided a comparative monomer yield (47%) to that generated by FA (56%).

In our prior studies, staining of the boron trihalide-treated lignocellulose sample with colorimetric dye, and further characterization of the polysaccharide extractives, gave high confidence that the majority of the cellulose and hemicellulose were removed from the lignocellulose material.^12^ Nevertheless, the structural details of the lignin resulting from this treatment protocol remained unclear. Herein, we provide further investigation into the degree of condensation/cross-linking of our lignin, which will now be referred to as “boron lignin,” as well as more accurately quantify the efficiency of polysaccharide removal. We compare this boron lignin to two different lignin samples generated from known protocols: (1) one obtained using the Klason method, which is known to produce some of the most highly condensed lignin, and (2) one generated from a formaldehyde (FA) treatment method by Shuai and Luterbacher (Figure 1).^32^ The Shuai–Luterbacher method is often considered the state-of-the-art for generating low condensed lignin. This low level of cross-linking is achieved by using formylating conditions, which modify the lignin structure and block nucleophilic sites that are necessary to form new C–C bonds. Notably, the use of alternative aldehydes or boronic acids in place of formic acid prevents this formylation, with slightly decreased monomer yields.^33^

The two lignin samples, Klason and FA lignin, survey the highest and lowest condensation levels that can be expected from lignin enrichments. As such, we sought to use these two methods to benchmark our analyses of lignin produced through our boron-mediated protocol. An overview of these results is summarized in Figure 1.

## Results and Discussion

In this study, two types of woods (white pine, a softwood, and beechwood, a hardwood) were used as the source of lignocellulose to evaluate the characteristics of the lignin generated. White pine sawdust contains ∼15–30 wt % of softwood lignin, and beechwood contains 21–31 wt % of hardwood lignin.^34^ When performing a lignin composition assessment, we find that our white pine samples contain 27.2 wt % insoluble lignin and our beechwood samples contain 22.6 wt % insoluble lignin (see Table 1, entry 1).^35^

**Table 1:** Lignin Extraction Efficiencies Using Three Different Approaches

entry	method	% of original mass (wt %)	carbohydrates remaining (wt %)
1	Klason	27.2^a^, 22.6^b^	0^a^^,^^b^
2	FA	82.1^a^, 81.9^b^	80^a^, 74^b^
3	boron	27.2^a^, 24.4^b^	15^a^, 20^b^

a White pine sawdust.

b Beechwood sawdust.^12,32^ % of original mass is the wt % of lignin mass as compared to starting masses of extractives-free lignocellulose. Carbohydrates remaining refers to the % of carbohydrate that is still in the lignin sample. This was assessed by first removing residual carbohydrates by performing a Klason treatment on each lignin sample. The mass decrease from this process was converted into carbohydrates remaining by the following conversion: (mg decrease/mg total sugars) × 100. The mg total sugars is the value obtained from the Klason treatment of extractives-free sawdust, which was assessed as 364 mg per 500 mg sample of white pine and 387 mg per 500 mg sample for beechwood.

The weight percentages and carbohydrate assessments obtained from all three extracted lignin samples are shown in Table 1. Carbohydrate assessment involved treating the FA lignin and boron lignin to a Klason extraction, which removes any remaining sugar from those lignin samples;^36^ the remaining solids are acid-insoluble lignins. For white pine samples, the acid-solubilized fraction was assessed for cellulose and hemicellulose percentages based on monosaccharide analysis (see Supporting Information for details).

The FA lignin protocol resulted in residual wood biomasses for both hardwood and softwood samples that are higher than expected, ∼82% (entry 2). This higher lignin mass is due to two reasons. First, the protocol introduces formyl groups on the nucleophilic aromatic sites of the lignin structure during the extraction process, adding molecular mass. Second, the protocol results in the derivatization and incomplete removal of polysaccharides.^32^ FA lignin (29 wt %) was determined to be acid-insoluble lignin based on Klason treatment. Sugar composition analysis (% cellulose and hemicellulose) for the FA lignin was complicated by the known sugar modifications (e.g., formation of diformylxylose) caused by FA treatment;^32^ over half of the sugars from the FA lignin sample could not be assigned as glucose, xylose, galactose, or mannose. However, Shuai and co-workers reported that for beechwood and poplar samples, nearly all hemicellulose is retained in the FA lignin, with the cellulose being the main component removed.^32^

Comparatively, the residual wood biomass weights following boron Lewis acid treatment for hardwood and softwood (entry 3) were in the range expected. However, while the wt % of boron lignin is comparable to Klason lignin (27.2 and 24.4%), subsequent Klason treatments suggest that 15–20% of carbohydrates remain. For white pine, the remaining sugars are 27% and 12% of the lignin weight for cellulose and hemicellulose, respectively. The acid-insoluble mass was 60 wt % of the boron lignin mass. Collectively, these data indicate a concomitant loss of ∼14% lignin during the extraction protocol.

Many literature reports have noted a distinct relationship between color and the degree of degradation in lignin samples. Specifically, a darker color is observed when quinones or conjugated aromatic structures are generated.^32^ The color observed in boron lignin is similar to that of FA lignin, qualitatively suggesting that boron lignin may possess a low level of condensation (see Supporting Information).

To quantitatively evaluate lignin condensation in our extracted lignin using boron trihalides, we subjected the boron lignin to a ruthenium-catalyzed hydrogenolysis to depolymerize the lignin at elevated pressure and temperature. The efficiency of hydrogenative depolymerization to generate aromatic monomers is directly reflective of the degree of cross-linking. High degrees of condensation limit monomer recovery from hydrogenolysis since the C–C cross-links are resistant to cleavage under these conditions.

However, it is worth noting that FA lignin is largely soluble in the hydrogenolysis solvent while Klason and boron lignin are mostly insoluble. While there is likely some solubilized lignin in all cases since the hydrogenolysis proceeds forward for all lignin samples, the level of solubility will undoubtedly affect the hydrogenolysis efficiency. Depolymerization of a partially soluble lignin source will be less efficient than that of a fully solubilized lignin source (leading to lower monomer yield). Attempts to solubilize boron lignin through acetylation and silylation methods were largely unsuccessful (see Supporting Information).^37,38^ As such, while the subsequent monomer analysis is insightful in a broad sense, a strict comparison between FA lignin and boron lignin cannot be made with this assay.

The presence of boric acid and boronic acids have been noted to affect depolymerization efficiencies due to their coordination to diols in the lignin structure.^33,39,40^ Boric acid is produced as a byproduct in the preparation of our boron lignin. However, the boric acid is separated from the lignin in this protocol by sequestration to the aqueous layer, and NMR studies indicate no observable boron retained in the lignin sample.

**Figure 2:**
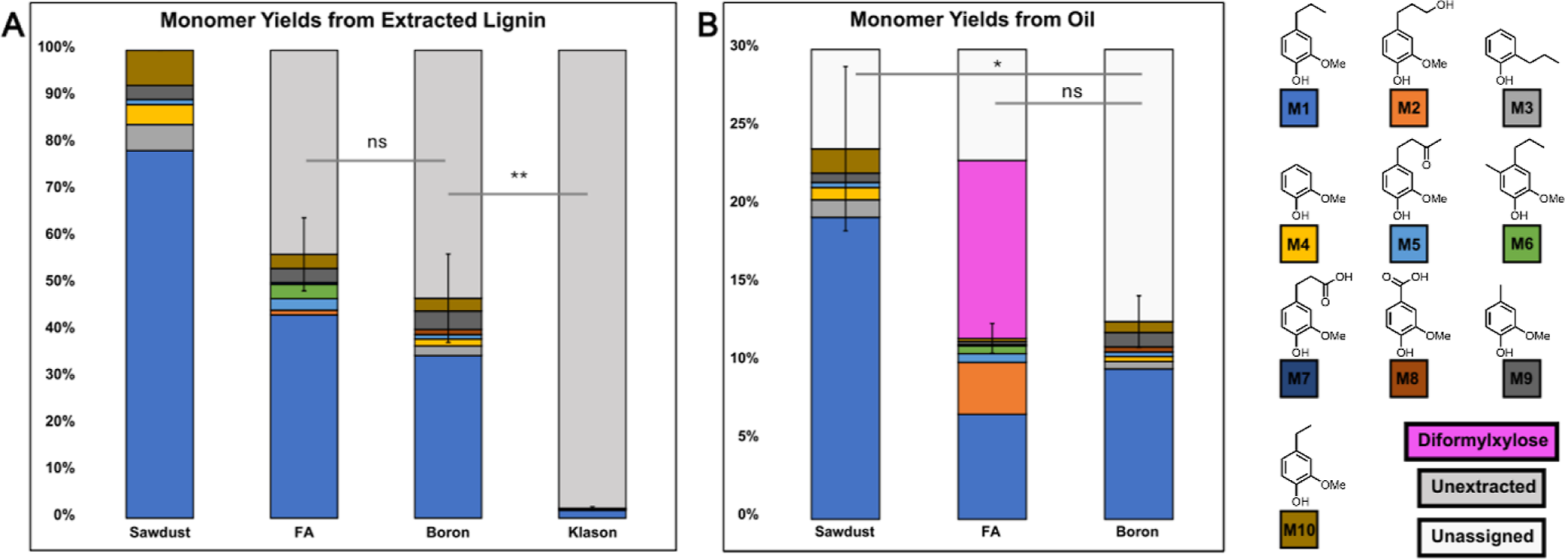
Monomer yields from lignin extracted from white pine sawdust using three different approaches. Monomer yields obtained after hydrogenolysis (optimal conditions, see Supporting Information) of extractives-free sawdust and lignin extracted by three different methods (FA, boron, Klason) are shown.^12,32^ (A) Monomer yields based on extracted lignin from extractives-free white pine sawdust. The highest monomer yield able to be obtained from white pine sawdust (24%) via “lignin first” strategy is shown for comparison. Thus, 24% = 100% as it is the maximum yield of cleavable monomers from the biomass. (B) Monomer yield based on hydrogenated oil (white pine). Yields shown are the average of three samples; student’s *t*-test was used for statistical comparison (*p < 0.05, **p < 0.01, ns = not significant).

We subjected all three lignin samples originating from extractives-free white pine sawdust to hydrogenolysis to identify and quantify the resulting aromatic monomers. Identical conditions and protocol for the hydrogenolysis were used for all lignin samples to maximize comparability (see Supporting Information for details). As expected, a low mass of monomer-rich oil (3 mg) was obtained after hydrogenolysis of Klason lignin (100 mg). Alternatively, higher oil masses were obtained after hydrogenolysis of sawdust alone (24 mg), boron lignin (25 mg), and FA lignin (36 mg). When analyzed by GC-FID, seven lignin monomers were identified from the white pine samples, with 2-methoxy-4-propylphenol (**M1**) constituting the majority of the resulting oil for all three lignin samples (Figure 2A). Details regarding monomer identification and quantification can be found in the Supporting Information. For FA lignin, one additional methylated monomer, 2-methoxy-5-methyl-4-propylphenol (**M6**), was identified, indicative of native structure formylation and reduction. In the “lignin first” comparison, where we subjected the extractives-free sawdust sample directly to our hydrogenolysis reaction, we observed the highest expected monomer yield (24%, the theoretical maximum percent of cleavable monomers in the softwood). Normalizing this result to 100, we find that Klason lignin provided 2% of extractable monomers, boron lignin provided 47%, and FA lignin provided 56% using our hydrogenative protocol (Figure 2A). These monomer yields are consistent with previous reported yields of softwood lignin, which have higher native levels of C–C linkages compared to hardwoods like beechwood.^41^ Looking at the mass ratio (mass of monomers obtained/mass of lignin extracted from biomass), this results in a 13% overall yield for FA lignin, 11% for boron lignin, and 0.5% for Klason lignin.

We also examined the mass ratio of identified monomers to the total mass of oil from hydrogenation and found that 12.6% of the oil from boron lignin consisted of identifiable monomers, comparable to that of FA lignin (11.5%) (Figure 2B). Importantly, 11.4% of the FA lignin oil consists of diformylxylose, reflective of the less efficient separation of polysaccharides, noted in Table 1.

**Figure 3:**
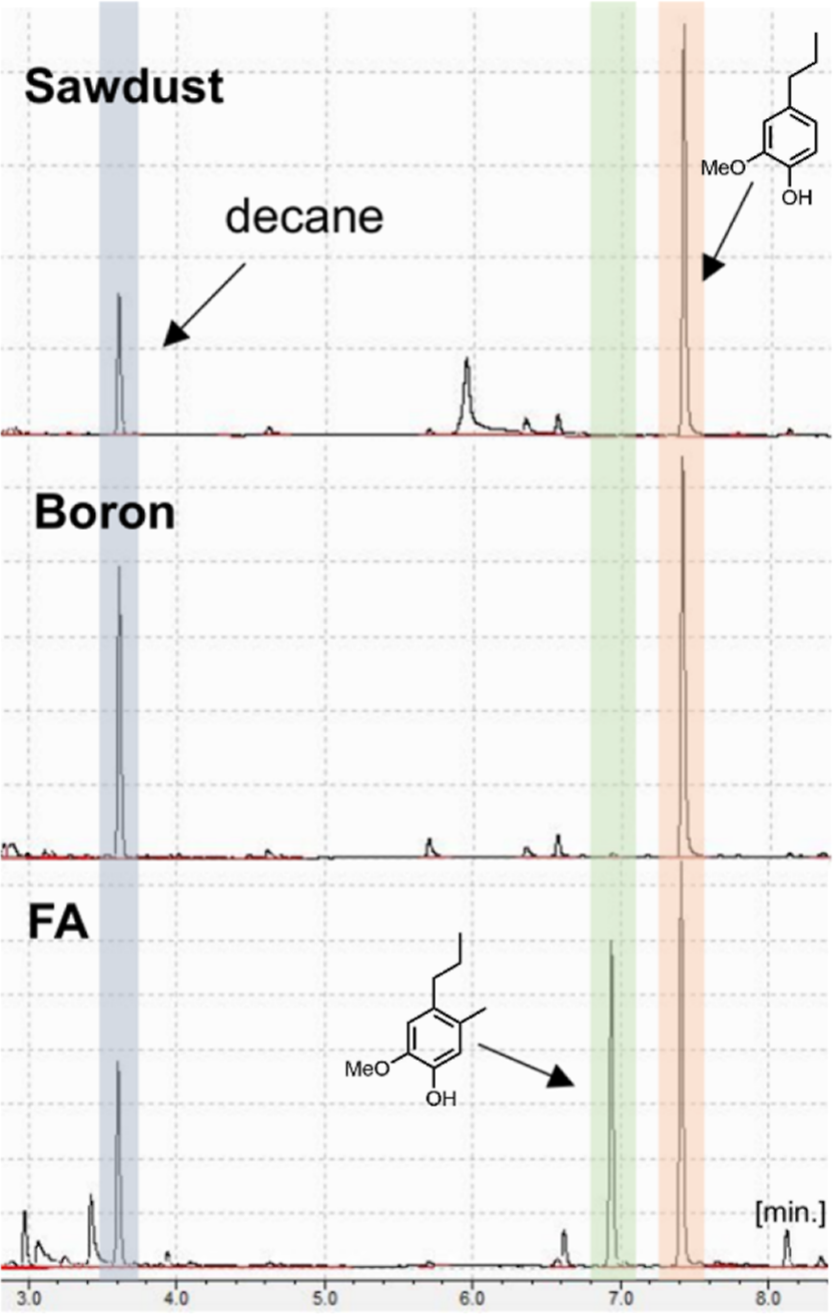
GC-FID of hydrogenated oil. GC-FID of monomer-rich oil obtained after hydrogenolysis of extractives-free white pine sawdust, boron lignin, and FA lignin is shown. Decane (gray) was used as an internal standard (ISTD) to quantify % yield of monomers. Propyl guaiacol is present in the sawdust and boron lignin hydrogenated oil (orange), whereas methylated propyl guaiacol is present only in the FA lignin hydrogenated oil (green).

Looking closer at the GC-FID traces of oil samples following hydrogenolysis (Figure 3), the peak distribution provides some evidence that the boron lignin protocol results in low structure modification compared to FA lignin. The peak pattern from the hydrogenated boron lignin is quite similar to that obtained from sawdust alone. The most abundant monomer observed after hydrogenolysis of white pine sawdust is propyl guaiacol, which is present in all three oils (orange). Methylated propyl guaiacol is only present in FA lignin (green).

**Figure 4:**
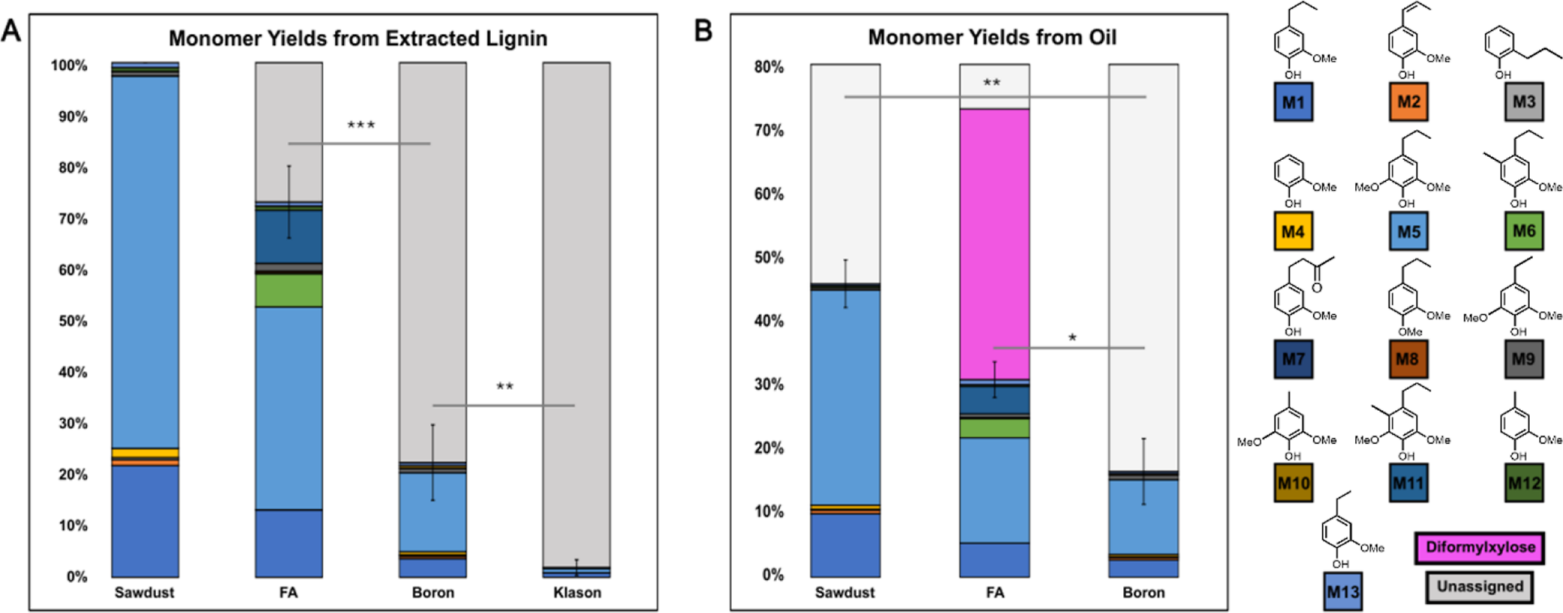
Monomer yields from lignin extracted from beechwood using three different approaches. Monomer yields obtained after hydrogenolysis (optimal conditions, see Supporting Information) of extractives-free beechwood sawdust and lignin extracted by three different methods (Klason, FA, boron) are shown.^12,32^ (A) Monomer yield based on extracted lignin. The maximum yield of cleavable monomers from biomass is 52%. Thus, 52% = 100%. (B) Monomer yield based on hydrogenated oil (beechwood). Yields shown are the average of three samples; student’s *t*-test was used for statistical comparison (*p < 0.05, **p < 0.01, ***p < 0.005).

We next tested the boron Lewis acid extraction along with the Klason and FA protocol on beechwood, a hardwood. The resulting lignin samples were also subjected to hydrogenolysis (Figure 4A). The trends in oil masses remained consistent with those of white pine (100 mg). The oil mass for Klason lignin was the lowest (8 mg) compared to FA lignin (38 mg), boron lignin (24 mg), and extractives-free beechwood sawdust (25 mg). For FA lignin, nine monomers were observed with 2-methoxy-4-propylphenol (**M1**) accounting for a large portion of the sample. However, 2,6-dimethoxy-4-propylphenol (**M5**) was the major constituent. Additionally, methylated monomers 2-methoxy-5-methyl-4-propylphenol (**M6**) and 2,6-dimethoxy-5-methyl-4-propylphenol (**M11**), as well as formylated monomer 4-hydroxy-3-methoxy-benzenepropanoic acid (**M7**) also contributed to the overall yield (38%). With the “lignin first” hydrogenation of extractives-free sawdust, 52% of the biomass was converted into identifiable monomers. Normalizing 52% to be the theoretical maximum yield meant the FA protocol provided 73% of expected cleavable monomers, consistent with previous reports.^32^ As expected, a low monomer yield was achieved from Klason lignin (2%) with **M1** contributing to over half the yield.

Here we observed a unique response when the boron lignin sample was manipulated after hydrogenolysis: a strong color change of the oil was observed within 5 min of exposure to air (vide infra). Further, monomer yields for this sample were surprisingly low. For boron lignin extracted from beechwood, eight total monomers were identified with 2,6-dimethoxy-4-propylphenol (**M5**, 15%) contributing the most to the overall yield (22%). **M1** contributed the second largest amount to the overall yield (4%). All monomers observed in boron lignin were also observed in beechwood sawdust.

**Scheme 1:**
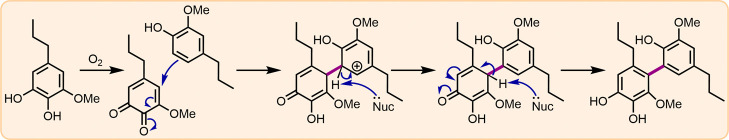
Proposed Mechanism for Monomer Dimerization and Pseudo-dimerization

We hypothesize that decomposition of boron lignin through posthydrogenolysis coupling ultimately resulted in low monomer yield. Boron Lewis acids, particularly BBr_3_, are known to rapidly cleave methyl aryl ethers.^42,43^ We therefore expect that the boron lignin would have a disproportionate amount of free phenolic –OH groups as compared to other lignin samples. Indeed, this supposition is supported by our spectroscopic analysis of boron lignin (vide infra). It is well documented that electron-rich phenols undergo spontaneous aerobic oxidation.^44^ For example, 2-hydroxy-3-methoxyphenol, a commercial reagent, is known to be highly air sensitive. Therefore, we anticipate that degradation of these demethylated monomers occurs through an aerobic oxidation, generating highly electrophilic quinones which undergo rapid C–C bond formation with remaining unoxidized monomers (see Scheme 1). This process would be accelerated in syringyl monomers (highly prevalent in beechwood) as compared to guaiacyl monomers (nearly 100% of the white pine lignin monomers) or coumaryl monomers, which would explain the differential behavior of our white pine and beechwood samples. We suspect that the dimerization occurs primarily after hydrogenolysis due to (a) the color change response, (b) the observation of particulate formation and precipitation from the sample during postreaction manipulation and workup, and (c) because the mass of oil obtained from the hydrogenolysis is high; cross-linking should limit the depolymerization process, resulting in low oil recovery as observed with Klason lignin samples.

**Figure 5:**
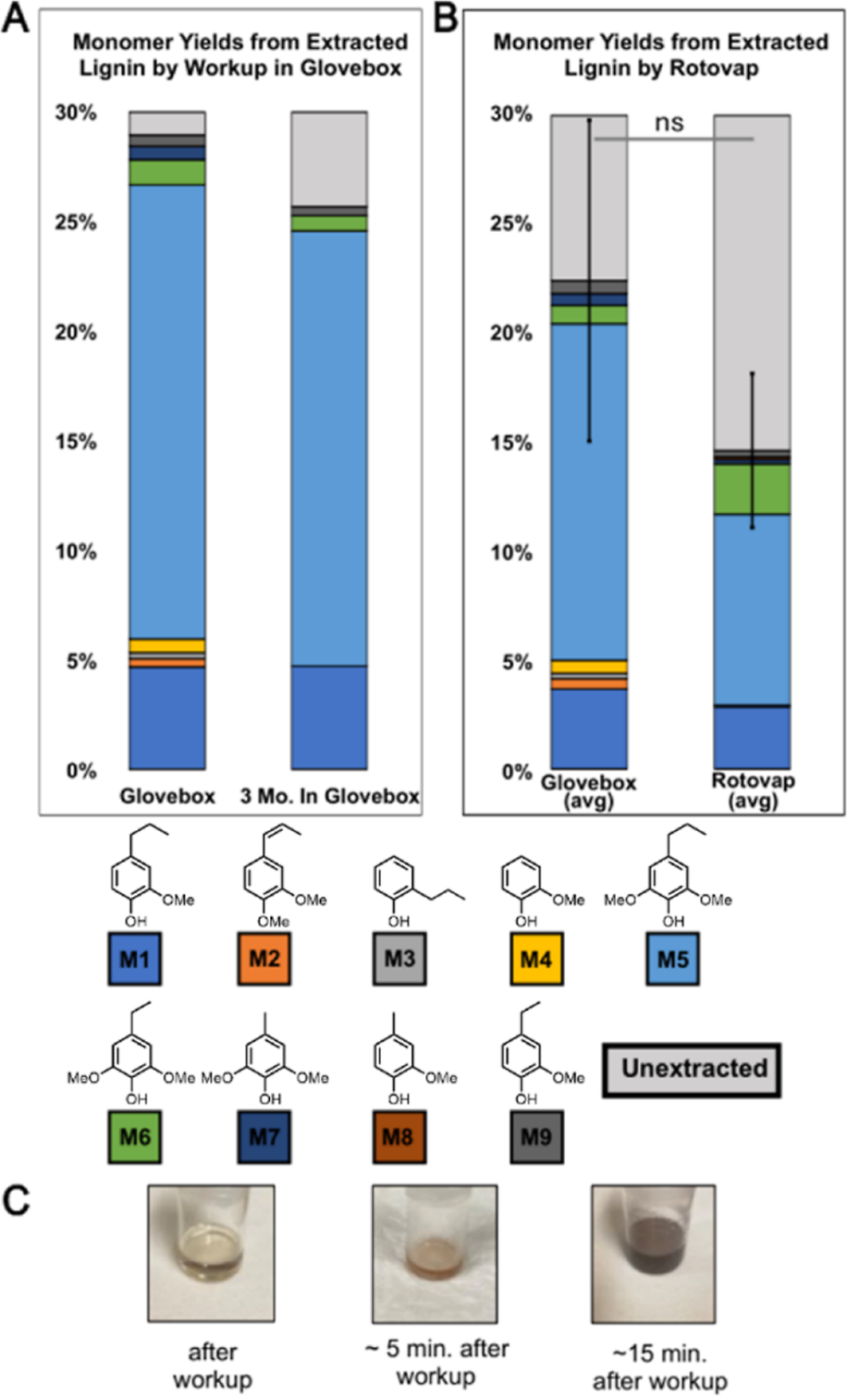
(A) Monomer yields of boron lignin extracted from beechwood following immediate transfer to an inert atmosphere glovebox without concentration and after storage of the same sample for 3 months. (B) Monomer yields of boron lignin extracted from beechwood following immediate transfer to an inert atmosphere glovebox without concentration, and for samples that were concentrated with rotary evaporation. Both preparations were performed in triplicate. (C) Progressive monomer degradation of the beechwood monomer oil upon exposure to air. Longer exposure time resulted in a strong color change from a pale yellow after workup to a light pink 5 min after workup to a dark brown 15 min after workup. The color change was accompanied with the formation of insoluble particulates. Yields shown are the average of three samples; student’s *t*-test was used for statistical comparison. ns = not significant.

To provide further evidence that degradation of the oil sample occurs at this late stage, we evaluated monomer levels when handling the hydrogenated oil sample in ambient air, in a glovebox with inert atmosphere, and after 3 months of storage in a glovebox (Figure 5). When following the same sample over time, the observed monomer yield is 3% lower (an 11% decrease from the original 28.9%) and monomer diversity is lessened (Figure 5A). **M2–M4** are no longer present in the 3 month aged sample. When workup was performed on a benchtop and the hydrogenated oil was dried under rotary evaporation (our typical protocol), the monomer yield was lower on average (14.5 ± 3.5%) compared to when workup was performed in a glovebox and the oil was not concentrated down (22.3 ± 7.3%) (Figure 5B). While this difference is not statistically significant due to the variability of the glovebox stored samples, monomer diversity is again affected, with **M2–M4** missing in the samples exposed to ambient air and subjected to rotary evaporation. The apparent trend of degradation over time would be consistent with the proposed air-mediated oxidation/cross-linking as a mechanism for degradation.

Ultimately, concentration of the oil upon rotary evaporation, whether from a sample kept in a glovebox or a sample on the benchtop, generated insoluble particulates and resulted in a strong color change (Figure 5C). The sensitivity to concentration is also consistent with degradation through coupling, as dimerization and polymerization reactions are significantly accelerated (and often performed) in concentrated solutions.^45^ Further investigation into the mechanistic details of this degradation is ongoing.

In line with the monomer yields, the mass percentage of identified monomers in the extracted oil for boron lignin (14%) is lower than that of FA lignin (27%) (Figure 4B). Similarly to white pine, a considerable percentage of the total FA lignin oil contains diformylxylose (37%).

### NMR Analysis

Along with GC analysis, the lignin samples extracted from white pine were subjected to 1D and 2D NMR analyses (see Supporting Information). White pine lignin was used for these analyses due to white pine lignin’s structural simplicity in comparison to beechwood; white pine is composed mainly of guaiacyl units while beechwood has a mixture of guaiacyl and syringyl units.^46,47^

**Figure 6:**
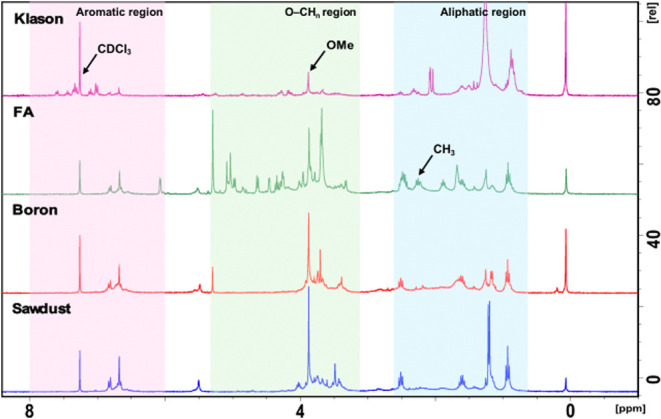
^1^H NMR of hydrogenated oil. ^1^H NMR of the monomeric oil obtained after hydrogenolysis of lignin (Klason, FA, boron) and sawdust is shown. The aromatic region (pink), a middle region where hydrogens neighboring oxygen centers appear (O–CH_*n*_ region) (green), and aliphatic region (blue) are highlighted. The diagnostic methoxy peak (3.9 ppm) belonging to propyl guaiacol and the methyl peak attributed to methylated propyl guaiacol in FA lignin oil (2.2 ppm) are labeled. White pine sawdust was used for this analysis.

The ^1^H NMR spectra of the oil obtained after hydrogenolysis of boron lignin, FA lignin, and Klason lignin were compared to that of extractives-free white pine sawdust to analyze the degree of monomer structure modification (Figure 6). Three regions are highlighted: the aromatic region (6–8 ppm), an O–CH_*n*_ region, which is where carbohydrates and methoxy groups appear (3.6–5.3 ppm), and an aliphatic region (0.9–1.6 ppm). All four samples showed evidence of the most abundant monomer, propyl guaiacol (diagnostic peaks at 6.7 and 6.8 ppm in the aromatic region, a singlet at 3.9 ppm for the methoxy methyl, and finally two triplets at 0.9 and 2.6 ppm and a sextet at 1.6 ppm). For Klason lignin oil, the highest variability was observed in the aromatic region, with peaks above 7 ppm; this is attributed to sulfation and cross-linking promoted by the high concentrations of sulfuric acid used, leading to downfield shifts of these aromatic protons.^48^ Additional peaks in the O–CH_*n*_ region (∼4.8 ppm) are reflective of alkene protons, generated by dehydration reactions.^49^ In the aliphatic region, the strong peaks around 0.9 and 1.2 ppm are attributed to grease contamination of the sample since these conditions are not reducing and therefore not expected to increase aliphatic signals relative to aromatic signals. The ^1^H NMR spectrum of the FA lignin oil is most complex in the O–CH_*n*_ region (green). This is largely due to signals arising from diformylxylose (5.09, 5.03, 4.97, 4.63, 4.46, 4.25, and 3.85–3.96 ppm), a xylose derivative formed during the FA lignin extraction protocol. In the aliphatic region, a number of additional peaks also arise, which are likely formylation products. One such anticipated product, methylated propyl guaiacol, is observed only in the FA oil (2.2 ppm for the methyl peak).^32^ Finally, boron lignin and extractives-free sawdust show minimal differences. Two significant changes are an observed decrease in peaks in the O–CH_*n*_ region for boron lignin, which arises from the lower compositional percent of polysaccharides, and a decrease in the quartet at 1.2 ppm, which remains unassigned. The high similarity of boron lignin and extractives-free sawdust indicates significant retention of native lignin structure.

**Figure 7:**
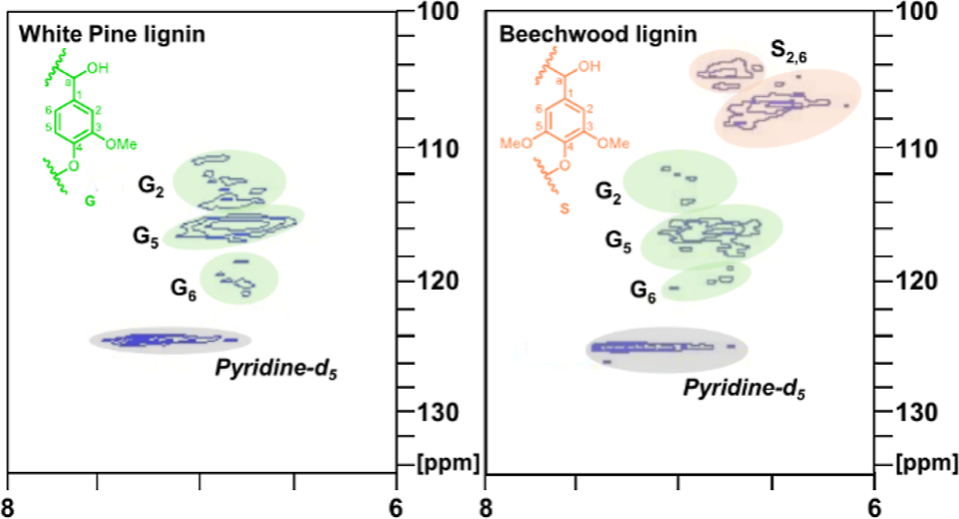
Aromatic regions in 2D HSQC spectra of boron lignin generated from white pine sawdust (left) and beechwood sawdust (right). The boron lignin generated from beechwood possesses a high content of syringyl *and* guaiacyl units while boron lignin generated from white pine only possesses a high content of guaiacyl units.

Because of the challenge of solubilizing the boron lignin generated from both white pine and beechwood sawdust, the solids were swelled and subjected to HSQC analysis as a gel following addition of pyridine-*d*_5_ and DMSO-*d*_6_.^50^ Despite this useful technique, the lignin aliphatic region did not resolve sufficiently for conclusive analysis (see Supporting Information for the full HSQC spectra). The aromatic region, however, did show distinct signals. For boron lignin generated from white pine, the **G**_**2**_, **G**_**5**_, and **G**_**6**_ protons can be observed (shown in Figure 7).^51^ For boron lignin from beechwood, the same **G**_**2**_, **G**_**5**_, and **G**_**6**_ protons are present as well as expected new syringyl protons assigned as **S**_**2,6**_. The presence of all expected protons in the aromatic region, especially nucleophilic positions **G**_**5**_ and **G**_**6**_, indicates that cross-linking is not extensive.^51^

**Figure 8:**
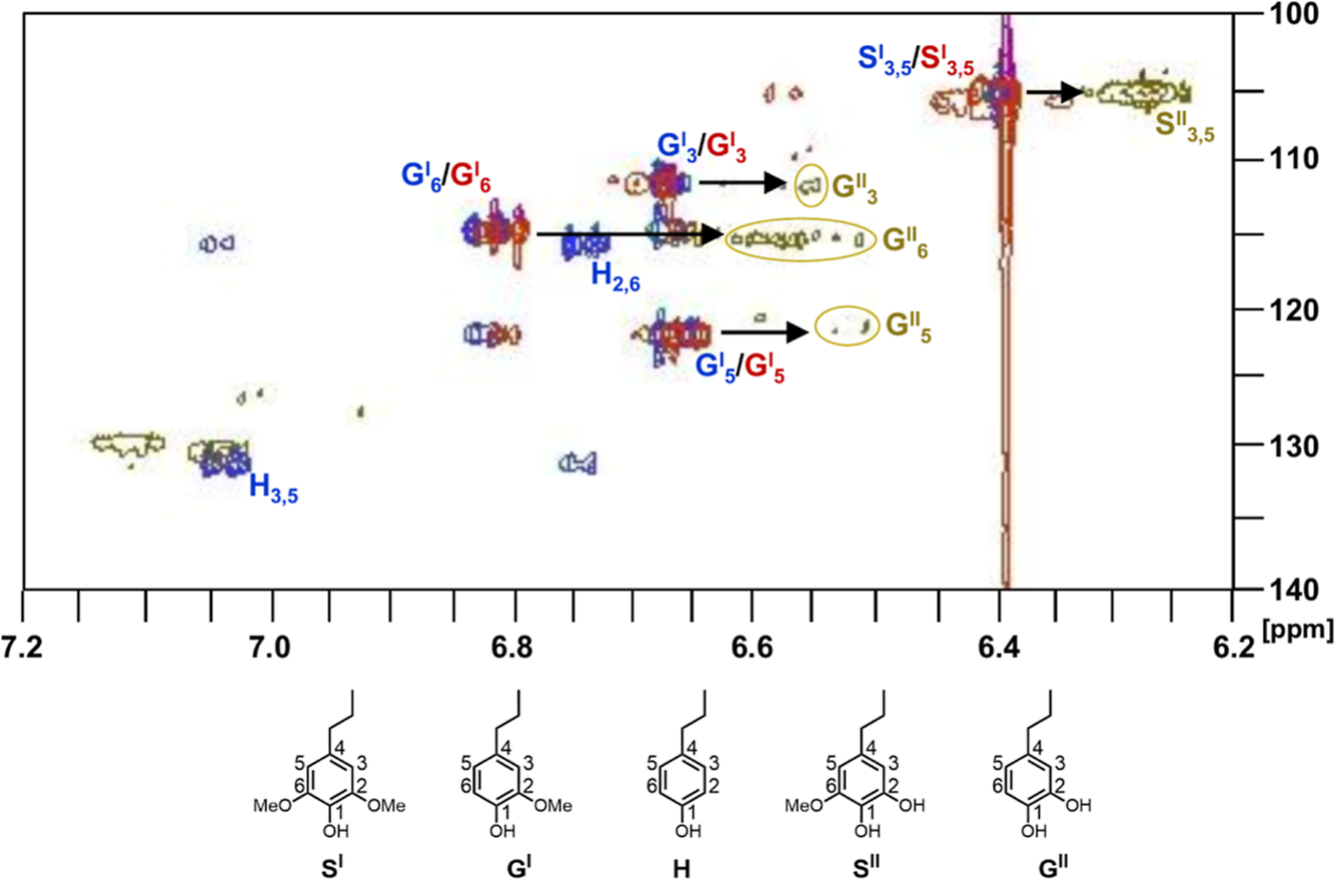
Aromatic regions in 2D HSQC spectra of hydrogenated oil of beechwood sawdust (red), a mixture of commercial guaiacyl, syringyl, and coumaryl monomers (blue), and hydrogenated oil of boron lignin (tan). 2-Methoxy-4-propylphenol (**G**^**I**^) and 2,6-dimethoxy-4-propylphenol (**S**^**I**^) are observed in both the commercial monomer mixture (blue spectrum) and the beechwood sawdust oil sample (red spectrum), while their demethylated derivatives (**G**^**II**^, **S**^**II**^) are present in the boron lignin oil sample (tan spectrum).

To further investigate the hypothesis that the boron Lewis acid treatment may result in demethylation, leading to monomers which are not air stable, an HSQC was obtained of the resulting oil after hydrogenolysis of boron lignin generated from beechwood (Figure 8). The spectrum (shown in tan) was compared to a mixture of each type of monomer (guaiacyl, syringyl, coumaryl) bought commercially (shown in blue) and to the oil from a hydrogenated beechwood sawdust sample (shown in red). The protons attributed to the syringyl and guaiacyl monomers in the red and blue spectra overlap with the sawdust peaks, confirming that these are the monomers present in the sawdust-derived oil sample. The coumaryl monomer was not observed in the sawdust and boron lignin oils. This is expected and these protons also did not appear in the lignin polymer HSQC (see Figure 7). Syringyl protons **S**_**3,5**_^**II**^ (∼6.28/105 ppm) and guaiacyl protons (**G**_**3**_^**II**^ 6.56/111 ppm, **G**_**5**_^**II**^ 6.51/120 ppm, and **G**_**6**_^**II**^ 6.61/115 ppm) from the boron lignin oil are shifted upfield compared to the commercial monomer mixture and the sawdust oil sample (**S**_**3,5**_^**I**^ 6.39/105 ppm, **G**_**3**_^**I**^ 6.68/111 ppm, **G**_**5**_^**I**^ 6.65/121 ppm, and **G**_**6**_^**I**^ 6.80/114 ppm). This is the expected shift if demethylation of the monomer has occurred, since electron density at these positions increases, effectively shielding the protons. Further, protons in the region 6.5–6.78/111–120 ppm (**G**_**3**_^**II**^, **G**_**5**_^**II**^ and **G**_**6**_^**II**^) match literature reported chemical shifts for 4-propylcatechol (**G**^**II**^).^52^ This aligns with our speculation regarding the demethylating potential of the boron Lewis acids used to generate boron lignin.

**Figure 9:**
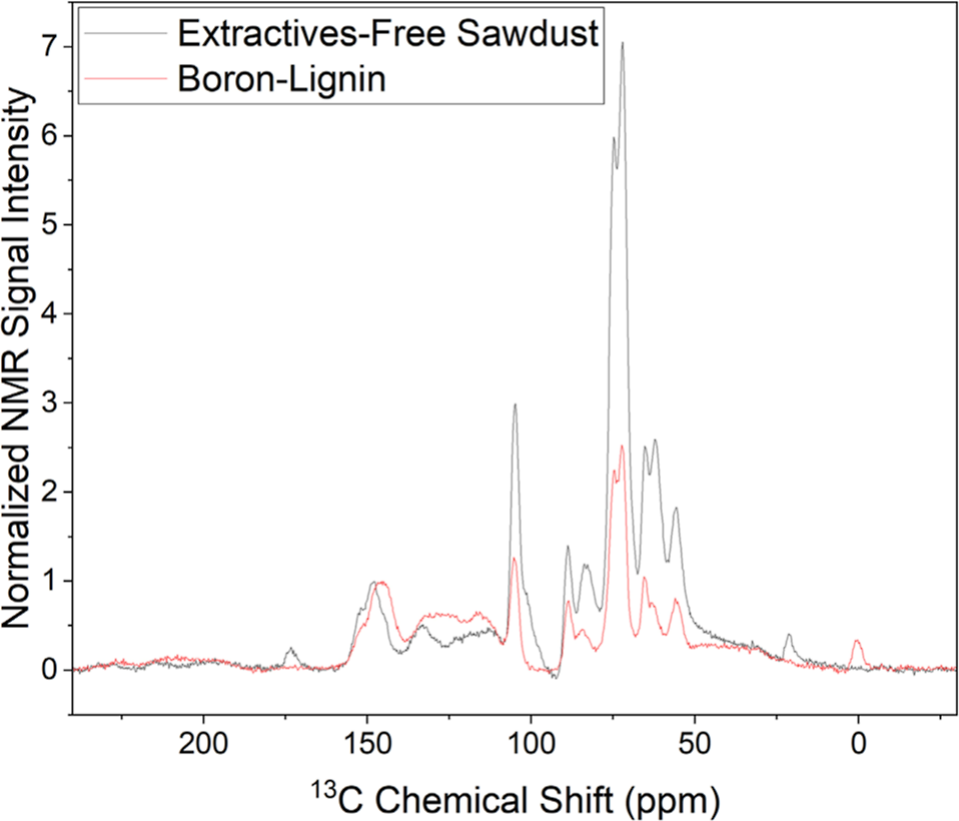
Normalized solid-state ^13^C multi-CP NMR spectra of boron lignin (red) and extractives-free white pine sawdust (black). The aromatic region is matched in the overlay since the lignin content is minimally changed during the boron lignin separation process. Relative to this, the intensity of the peaks attributed to cellulose substantially decreased in the boron lignin sample compared to that of the sawdust sample (52–66%). The same trend is true for the methoxy carbons in the boron lignin spectrum, where a 52% decrease is observed compared to the same peaks in the sawdust spectrum, providing further evidence of demethylation during separation.

Furthermore, cross-polarization by multiple contact periods (Multi-CP) was used to obtain quantitative ^13^C solid-state NMR spectra of boron lignin and extractives-free white pine sawdust (lignocellulose) shown in Figure 9. The largest aromatic peak (∼145 ppm) for lignin and sawdust were matched in intensity in the overlay and normalized to 1.^53^ Relative to the aromatic region, the peaks associated more strictly to cellulose (∼105 and ∼89 ppm) decreased by 66% (58% and 52%, respectively).^54,55^ The peak associated with the carbons of methoxy groups (∼56 ppm) also decreased substantially (52%), which is consistent with our prior data suggesting demethylation during treatment with boron trihalides.^56−59^

## Conclusions

Collectively, our results are consistent with a low-condensed lignin being generated through boron Lewis acid separation, with fairly good sugar removal (∼85%). It is notable that the boron lignin samples provided 47% of the expected monomers following hydrogenolysis despite being largely insoluble in the hydrogenolysis conditions. This result compares surprisingly well to the 56% monomer yield obtained from FA lignin, which is fully soluble and more efficiently depolymerized. Nevertheless, the condensation degree cannot be fully benchmarked to FA lignin or other common/commercial lignin sources because of these solubility differences. Therefore, solubilizing boron lignin remains an area of future work to properly evaluate condensation levels, to provide more efficient monomer production capacity, and to expand its general utility.

Additionally, the degradation of the hydrogenolysis sample from hardwood-sourced boron lignin (beechwood) was unexpected but gives insight into potential effects of boron Lewis acid treatment on the lignin scaffold. Our spectroscopic analyses provide support for the boron lignin preparation resulting in demethylation (solid state NMR and HSQC). This is consistent with a proposed degradation through aerobic oxidation of demethylated syringyl units, leading to monomer coupling following hydrogenolysis. To our knowledge, this type of postdepolymerization degradation has not been examined or noted in other lignin depolymerization settings. We will continue to investigate the mechanistic features of the monomer behavior, as well as ways to mitigate the postulated postdepolymerization reactivity. Future work will also examine alternate sources of hardwood to evaluate whether the degree of degradation correlates with the compositional percentage of syringyl subunits.

Spectroscopic evidence suggests limited alterations of the lignin structure by the boron Lewis acid extraction protocol, with high similarity of boron lignin to native lignin. This is a significant feature of the boron lignin method. Many commonly employed enrichment/separation methods introduce sulfur, oxidize or reduce the lignin framework, and eliminate alcohols (providing alkene products).^60^ While each of these lignins has important utility in both the academic and industrial setting, access to minimally altered lignin is still nontrivial. Accessing lignin-rich samples with native-like structure is of value to many, including plant biologists who study the biopolymers and materials chemists who compare and contrast the physical properties of different lignin sources. Finally, if a low level of structural alteration is occurring, as suggested by our analysis to date, then the potential remains to increase monomer yields from boron lignin significantly, with the ultimate goal of approaching a “lignin first” performance.

## Experimental Section

### Materials

All reactions were performed under air-free and water-free conditions unless otherwise stated. All deuterated solvents were stored over molecular sieves (4 Å). Methanol (99.9%, HPLC grade), sodium bicarbonate (Certified ACS), sodium sulfate (anhydrous, granular, certified ACS), tetrahydrofuran (THF, HPLC grade), and dimethylsulfoxide (DMSO, 99.9%, Certified ACS) were purchased from Fisher Chemical. Dichloromethane (CH_2_Cl_2_, Certified ACS stabilized, 99.5%) was purchased from Fisher Chemical and dried using a solvent system (LC Technology Solutions Inc.). All water used for experimentation was deionized (DI). Untreated white pine wood blocks (*Pinus strobus*) were purchased from Lowe’s and sanded into sawdust (particle size 4 μm, based on visualization using a Olympus IX71 inverted microscope, ×20 magnification). Untreated beechwood blocks (*Fagus sylvatica*) were purchased from Etsy (mgwooddekoration) and sanded into sawdust (particle size 6 μm). Ethanol (200 proof) was purchased from Koptec. Benzene (99.0%, ACS reagent), 1,4-dioxane (anhydrous, 99.8%), Kraft lignin, imidazole (anhydrous, free-flowing, Redi-Pri, ACS reagent, >99.0%), acetic anhydride (99.5%), *N*,*O*-bis(trimethylsilyl)trifluoroacetamide (BSTFA, 99.0%), acetone-*d*_6_ (99.9 atom % D), chloroform-*d* (CDCl_3_, 99.8 atom % D, contains 0.03% v/v TMS), and deuterated DMSO (DMSO-*d*_6_, 99.9 atom % D) were purchased from Sigma-Aldrich. Boron trichloride (BCl_3_, 1 M CH_2_Cl_2_) and boron tribromide (BBr_3_, 1 M CH_2_Cl_2_) were purchased from Sigma-Aldrich with a Sure/Seal. Formaldehyde (FA, 37.0% in aq. soln., ACS, 36.5–38.0% stab. with 10.0–15.0% methanol) and pyridine-*d*_5_ (99.5 atom % D) were purchased from Thermoscientific. Hydrochloric acid (HCl, 36.5–38.0%, ACS grade) and sulfuric acid (H_2_SO_4_, 95.0–98.0%) were purchased from VWR Chemicals. Pyridine (99.0%) was purchased from Sigma-Aldrich, distilled, and stored over molecular sieves (4 Å) before use. Ruthenium on carbon (Ru/C, 5 wt % loading) was purchased from Strem Chemicals.

### Methods

#### Removal of Extractives from White Pine and Beechwood Sawdust

The procedure was followed exactly according to literature.^12^ Sawdust was transferred to a 100 mL round-bottom flask (RBF) containing a stir bar. A 1:2 mixture of ethanol/benzene (51.0 mL) was added to the RBF. The mixture was heated to reflux and stirred for 6 h, then filtered and washed with ethanol (20.0 mL). The solid residue was transferred to a 100 mL RBF containing a stir bar. Ethanol (50.0 mL) was added, and the mixture was heated to reflux and stirred for 4 h. The mixture was filtered and washed with water (50.0 mL), and the solid residue was transferred to a 250 mL RBF containing a stir bar. Water was added (120 mL), and the mixture was heated to reflux and stirred for 1 h. The mixture was filtered, washed with water (150 mL), and dried in a 90 °C oven for 1 h to afford extractives-free sawdust (3.28 g from white pine and 3.49 g from beechwood).

#### Boron Lignin Separation

The separation procedure was followed exactly according to literature for the two biomasses described (white pine, beechwood).^12^ Extractives-free sawdust (500 mg) was ground into fine powder and loaded in a 250 mL RBF containing a stir bar. While vigorously stirring, CH_2_Cl_2_ (30.0 mL) was added followed by BCl_3_ (8.00 mL, 1 M in CH_2_Cl_2_) and then BBr_3_ (8.00 mL, 1 M in CH_2_Cl_2_). The mixture was allowed to stir at room temperature for 18 h. The reaction mixture was quenched with water (30.0 mL) and filtered. The organic layer was separated and washed with water (3 × 30.0 mL), dried over Na_2_SO_4_, filtered, and dried by rotary evaporation. The solid residue was dried under high vacuum for 2 h and resubjected to the boron trihalide procedure another 4 times for white pine and 3 times for beechwood to afford boron lignin.

#### FA Lignin Extraction

The procedure was followed exactly according to literature, other than biomass source.^32^ Extractives-free sawdust (1.00 g) was transferred to a 50 mL RBF containing a stir bar. 1,4-Dioxane (9.00 mL), HCl (0.420 mL), and formaldehyde (1.00 mL) was added to the RBF. The RBF containing the reaction mixture was connected to a reflux condenser, heated to 80 °C, and stirred at 300 revolutions per minute (RPM) for 5 h. The mixture was filtered and washed with 1,4-dioxane until the filtrate was colorless. The filtrate was neutralized with a bicarbonate solution (∼420 mg in 5.00 mL water). The solvent was removed by a rotary evaporator at 60 °C. The dried residue was redissolved in THF to extract lignin and then was filtered, leaving salt and carbohydrates behind as precipitates. THF was removed by rotary evaporation at 40 °C. The resultant orange oil was dried under high vacuum overnight to afford FA lignin (821 mg for white pine, 819 mg for beechwood).

#### Klason Lignin Extraction

The extraction was performed following a modified literature procedure.^32^ Extractives-free sawdust (500 mg) was ground into fine powder and loaded into a 50 mL beaker followed by an addition of a 72.0 wt % H_2_SO_4_ solution (7.50 mL). The mixture was left at room temperature for 2 h and stirred with a glass rod every 10 min. The slurry was transferred to a 500 mL RBF with DI water (290 mL) and heated to reflux for 4 h. The resultant precipitate was filtered, washed with boiling water (30.0 mL), and air-dried overnight to afford Klason lignin from white pine (136 mg, 27.1 wt %) and beechwood (105 mg, 20.8 wt %).

#### Hydrogenolysis of Lignin

Lignin (100 mg) was ground into a fine powder and added to a 25 mL glass insert (custom-made, shown in Figure S3.6) containing a magnetic stir bar and degassed methanol (5.00 mL). The mixture was subjected to sonication (Branson, 2800) for 5 min or until it appeared as homogeneous as possible. Ru/C (40.0 mg) was added to the mixture. The glass insert was then sealed in a 50 mL pressure reactor (custom-made, shown in Figure S3.6) and purged 3 times with H_2_ at 200 pound-force per square inch (psi). The reactor was pressurized with H_2_ (380 psi) and heated to 220 °C with high-temperature heating tape (Omega) connected to a variable power supply controlled by a proportional, integral, and derivative (PID) temperature controller (Omega) with a K-type thermocouple that measured the reaction temperature through a steel thermowell. The reactor was held at 220 °C and stirred at 400 RPM for 24 h. After 24 h, the reactor was cooled to room temperature before releasing the pressure. The catalyst, along with remaining precipitates, were filtered and rinsed with CH_2_Cl_2_ (10.0 mL). The filtrate was concentrated by rotary evaporation to provide a monomer-rich oil, which was subsequently analyzed by GC–MS and GC-FID.

*Note:* the monomer oil resulting from hydrogenolysis of boron lignin originating from beechwood was not concentrated down before GC-FID analysis. Instead, the workup was performed in a glovebox where the filtrate was directly prepared as a 25.0 mL solution with CH_2_Cl_2_ whereas the other oils were redissolved in 5.00 mL CH_2_Cl_2_ after workup as described (vide infra).

#### Silylation of Boron Lignin

The procedure was conducted according to literature.^38^ Boron lignin (60.0 mg) was transferred to a 100 mL RBF containing a stir bar. BSTFA (60.0 mL) was added. The reaction mixture was heated to 80 °C and stirred vigorously for 1 h. The mass of resultant silylated boron lignin was 86.0 mg (60.0 mg insoluble, 26.0 mg soluble). Subsequent boron lignin ^1^H NMR analysis of the solubilized portion showed a complete absence of aromatic signals, which are present in lignin and lignin derivatives.

#### Acetylation of Boron Lignin

The procedure was conducted in accordance to literature.^37^ Boron lignin (51.0 mg) was dissolved in pyridine (0.204 mL) in a 5 mL RBF. Acetic anhydride (10.0 equiv) was added, and the reaction mixture was stirred at room temperature for 18 h. HCl (1%, 10 volumes) was added at 0 °C, and the resulting precipitate was filtered and washed with water to neutral pH. The acetylated lignin was dried in an oven (40 °C) overnight. The mass of resultant acetylated boron lignin was 41.0 mg (33.0 mg insoluble, 8.00 mg soluble). Subsequent ^1^H NMR analysis of the solubilized portion showed a complete absence of aromatic signals, which are present in lignin and lignin derivatives.

#### Boron Lignin Solubility Test

Boron lignin (54.0 mg) was dissolved in DMSO (3.00 mL) and sonicated for 2 days at 40 °C. The remaining solid was filtered through a glass filter paper under vacuum. The filtrate was concentrated via bulb-to-bulb transfer to obtain a dried residue (9.00 mg). The remaining solid residue was oven-dried (100 °C) overnight (42.0 mg). The procedure was repeated with DMSO-*d*_6_ (3.00 mL) and the filtrate was not concentrated down. Subsequent ^1^H NMR analysis of the solubilized portion showed a complete absence of aromatic signals, which are present in lignin and lignin derivatives.

#### Gel NMR (2D HSQC) Experiment of Extracted Lignin

The procedure is a modification of a reported protocol.^50^ To a 5 mm NMR tube was added boron lignin or FA lignin (30.0–60.0 mg). The lignin was evenly dispersed along the length of the horizontally positioned NMR tube. A 4:1 mixture DMSO-*d*_6_/pyridine-*d*_5_ (0.600 mL) was transferred into the NMR tube on the bottom and along the sides. The NMR tube was vortexed (700 RPM) using a digital vortex mixer (Fisher Scientific) for 5 min and sonicated for 2 days to make the mixture as homogeneous as possible. These samples were then analyzed by 2D NMR. Parameter details are reported in the Supporting Information.

## Supporting Information Available

Experimental procedures for lignin extraction, lignin content determination, sugar analyses; analytical procedures for NMR, 2D HSQC, GC–MS, GC-FID, HPLC; analysis results; all boron lignin separation results and biomass analysis (solid-state Multi-CP ^13^C NMR, GC–MS, GC-FID, NMR, 2D HSQC) (PDF)

## Abbreviations

HPLC: high performance liquid chromatography
HSQC: heteronuclear single quantum coherence spectroscopy
NMR: nuclear magnetic resonance
GC: gas chromatography
GC-FID: gas chromatography-flame ionization detector
GC–MS: gas chromatography–mass spectrometry
DMSO: dimethylsulfoxide
CDCl_3_: chloroform-*d*
ppm: parts per million
