# Supplementary material for: Boron Lewis Acid Extraction of Wood Generates High Quality Lignin
Source: ACS Sustain Chem Eng. 2024 Nov 14;12(47):17210–21. doi: 10.1021/acssuschemeng.4c06206 (PMC11600917; doi:10.1021/acssuschemeng.4c06206)
Supplement: Supplementary file 1 — sc4c06206_si_001.pdf [file sc4c06206_si_001.pdf]

Supporting Information

Boron Lewis Acid Extraction of Wood Generates High Quality Lignin

Theodora E. Leventis,[a] Patrick Judge,[b] Jialiang Zhang,[b] M. Zain H. Kazmi,[c] Marcus B. Foston,[b] and Florence J. Williams [*a]

[a]The University of Iowa, Iowa City, IA, United States 52242-1002
[b]Washington University in St. Louis, St. Louis, MO, United States 63130-4899
[c]University of Alberta, Edmonton, AB, Canada T6G 2R3

*Corresponding Author: Florence-Williams@uiowa.edu

S1. Materials and Methods S2

S1.1 Chemicals and Materials S2
S1.2 Experimental Methods S2
S1.3 Analytical Methods S5

S2. Supplementary Text S8

S2.1 Monomer Identification and Quantification S8

S3. Figures S3.1-S3.18 S10

S4. Tables S4.1-S4.4 S23

S5. References S28

S1. Materials and Methods

S1.1 Chemicals and Materials

All reactions were performed under air-free and water-free conditions unless otherwise stated. All deuterated solvents were stored over molecular sieves (4 Å). Methanol (99.9%, HPLC grade), sodium bicarbonate (Certified ACS), sodium sulfate (anhydrous, granular, Certified ACS), tetrahydrofuran (THF, HPLC grade), and dimethylsulfoxide (DMSO, 99.9%, Certified ACS) were purchased from Fisher Chemical. Dichloromethane (CH_2Cl_2, certified ACS stabilized, 99.5%) was purchased from Fisher Chemical and dried using a solvent system (LC Technology Solutions Inc.). All water used for experimentation was deionized (DI). Untreated white pine wood blocks (*Pinus strobus*) were purchased from Lowe's and sanded into sawdust (particle size 4 μm, based on visualization using a Olympus IX71 inverted microscope, x20 magnification). Untreated beechwood blocks (*Fagus sylvatica)* were purchased from Etsy (mgwooddekoration) and sanded into sawdust (particle size 6 μm). Ethanol (200 proof) was purchased from Koptec. Benzene (99.0%, ACS reagent), 1,4-dioxane (anhydrous, 99.8%), Kraft lignin, imidazole (anhydrous, free flowing, Redi-Pri^{TM}, ACS reagent, >99.0%), acetic anhydride (99.5%), *N,*O-Bis(trimethylsilyl)trifluoroacetamide (BSTFA, 99.0%), acetone-d_6 (99.9 atom % D), chloroform-*d* ($CDCl_3$, 99.8 atom % D, contains 0.03% v/v TMS), and deuterated DMSO (DMSO-d_6, 99.9 atom % D) were purchased from Sigma Aldrich. Boron trichloride (BCl_3, 1 M CH_2Cl_2) and boron tribromide (BBr_3, 1 M CH_2Cl_2) were purchased from Sigma Aldrich with a Sure/Seal^{TM}. Formaldehyde (FA, 37.0% in aq. soln., ACS, 36.5-38.0% stab. with 10.0-15.0% methanol) and pyridine-d_5 (99.5 atom % D) were purchased from Thermoscientific. Hydrochloric acid (HCl, 36.5-38.0%, ACS grade) and sulfuric acid (H_2SO_4, 95.0-98.0%) were purchased from VWR Chemicals. Pyridine (99.0%) was purchased from Sigma Aldrich, distilled, and stored over molecular sieves (4 Å) before use. Ruthenium on carbon (Ru/C, 5 wt% loading) was purchased from Strem Chemicals. 2-methoxy-4-propylphenol (99.7%), 2-methoxyphenol (99.8%), 1,2-dimethoxy-4-propenylbenzene (98.0%), 3-hydroxy-4-methoxybenzoic acid (97.0%), 4-hydroxy-3-methoxybenzenepropanoic acid (99.3%), 1,2-dimethoxy-4-propylbenzene (97.0%), 2-methoxy-4-methylphenol (98.0%), 2-methoxy-4-ethylphenol (98.0%), and 2,6-dimethoxy-4-propylphenol (98.0%) were purchased from AmBeed. 2,6-dimethoxy-4-ethylphenol (97.0%) and 2,6-dimethoxy-4-methylphenol (98.0%) were purchased from Aaron Chemicals Product List. 4-propylphenol (>99.0%) was purchased from TCI.

S1.2 Experimental Methods

S1.2.1 Removal of extractives from white pine and beechwood sawdust

The procedure was followed exactly according to literature for the two biomasses described (white pine, beechwood).[1] Sawdust was transferred to a 100 mL round bottom flask (RBF) containing a stir bar. A 1:2 mixture of ethanol/benzene (51.0 mL) was added to the RBF. The mixture was heated to reflux and stirred for 6 h, then filtered and washed with ethanol (20.0 mL). The solid residue was transferred to a 100 mL RBF containing a stir bar. Ethanol (50.0 mL) was added, and the mixture was heated to reflux and stirred for 4 h. The mixture was filtered and washed with water (50.0 mL). The solid residue was transferred to a 250 mL RBF containing a stir bar. Water was added (120. mL), and the mixture was heated to reflux and stirred for 1 h. The mixture was filtered, washed with water (150. mL), and dried in a 90 °C oven for 1 h to afford extractives-free sawdust (3.28 g from white pine and 3.49 g from beechwood).

S1.2.2 Boron lignin separation

The separation procedure was followed exactly according to literature for the two biomasses described (white pine, beechwood).[1] Extractives-free sawdust (500. mg) was ground into fine powder and loaded in a 250 mL RBF containing a stir bar. While vigorously stirring, CH_2Cl_2 (30.0 mL) was added followed by BCl_3 (8.00 mL, 1 M in CH_2Cl_2) and then BBr_3 (8.00 mL, 1 M in CH_2Cl_2). The mixture was allowed to stir at room temperature for 18 h. The reaction mixture was quenched with water (30.0 mL) and filtered. The organic layer was separated and washed with water (3 x 30.0 mL), dried over Na_2SO_4, filtered, and dried by rotary evaporation. The solid residue was dried under high vacuum for 2 h and resubjected to the boron trihalide procedure another 4 times for white pine and 3 times for beechwood to afford boron lignin.

The mass of solid residue and organic layer after each boron trihalide treatment is reported in **Figure S3.1**. Hydrogen nuclear magnetic resonance (^1H NMR) of the total organic layer (<5.00% of biomass) is reported in **Figures S3.2-S3.3**. Heteronuclear single quantum correlation (2D HSQC) of the lignin-rich solid is reported in **Figures S3.4-S3.5**. Note: quenching the boron trihalide mixture in a 0 °C bath did not significantly change mass of lignin or monomer yield.

S1.2.3 FA lignin extraction

The procedure was followed exactly according to literature, other than biomass source.[2] Extractives-free sawdust (1.00 g) was transferred to a 50 mL RBF containing a stir bar. 1,4-dioxane (9.00 mL), HCl (0.420 mL), and formaldehyde (1.00 mL) was added to the RBF. The RBF containing the reaction mixture was connected to a reflux condenser, heated to 80 °C, and stirred at 300 revolutions per minute (RPM) for 5 h. The mixture was filtered and washed with 1,4-dioxane until the filtrate was colorless. The filtrate was neutralized with a bicarbonate solution (~ 420. mg in 5.00 mL water). The solvent was removed by rotary evaporation at 60 °C. The dried residue was redissolved in THF to extract lignin and then was filtered, leaving salt and carbohydrates behind as precipitates. THF was removed by rotary evaporation at 40 °C. The resultant orange oil was dried on high vacuum overnight to afford FA lignin (821 mg for white pine, 819 mg for beechwood).

S1.2.4 Klason lignin extraction

The extraction was performed following a modified literature procedure.[2] Extractives-free sawdust (500. mg) was ground into fine powder and loaded into a 50 mL beaker with an addition of a 72.0 wt% H_2SO_4 solution (7.50 mL). The mixture was left at room temperature for 2 h and stirred with a glass rod every 10 min. The slurry was transferred to a 500 mL RBF with DI water (290. mL) and heated to reflux for 4 h. The resultant precipitate was filtered, washed with boiling water (30.0 mL), and air-dried overnight to afford Klason lignin from white pine (136 mg, 27.1 wt. %) and beechwood (105 mg, 20.8 wt. %). Klason lignin protocol on boron lignin and FA lignin resulted in the removal of remaining polysaccharides. The amount of sugar removed from each type of lignin is listed in **Table S4.1**.

S1.2.5 Hydrogenolysis of lignin

Lignin (100. mg) was ground into a fine powder and added to a 25 mL glass insert (custom-made, shown in **Figure S3.6**) containing a magnetic stir bar and degassed methanol (5.00 mL). The mixture was subjected to sonication (Branson, 2800) for 5 min, or until it appeared as homogenous as possible. Ru/C (40.0 mg) was added to the mixture. The glass insert was then sealed in a 50 mL pressure reactor (custom-made, shown in **Figure S3.6**) and purged 3 times with H_2 at 200 pound-force per square inch (psi). The reactor was pressurized with H_2 (380 psi) and heated to 220 °C with high-temperature heating tape (Omega) connected to a variable power

supply controlled by a proportional, integral, and derivative (PID) temperature controller (Omega) with a K-type thermocouple that measured the reaction temperature through a steel thermowell. The reactor was held at 220 °C and stirred at 400 RPM for 24 h. After 24 h, the reactor was cooled to room temperature before releasing the pressure. The catalyst, along with remaining precipitates, were filtered and rinsed with CH_2Cl_2 (10.0 mL). The filtrate was concentrated by rotary evaporation to provide a monomer-rich oil, which was subsequently analyzed by gas chromatography-mass spectrometry (GC-MS) and gas chromatography-flame ionization detector (GC-FID). An optimization table of selected hydrogenolysis reactions is listed in **Table S4.2**. FA lignin was chosen as the lignin to optimize with as the protocol produced a larger amount of lignin and had a shorter reaction time compared to the procedures in which boron and Klason lignin were generated.

Note: The monomer oil resulting from hydrogenolysis of boron lignin originating from beechwood was not concentrated down before GC-FID analysis. Instead, the workup was performed in a glovebox where the filtrate was directly prepared as a solution with CH_2Cl_2 (25.0 mL) whereas the other oils were redissolved in CH_2Cl_2 (5.00 mL) after workup as described (vide infra). Progressive monomer degradation of the beechwood monomer oil when exposed to air is shown in **Figure S3.7**.

2D HSQCs of the monomeric oil after hydrogenolysis of extractives-free beechwood sawdust and boron lignin originating from beechwood sawdust are listed in **Figures S3.8-S3.9**. ^{1}H NMRs of the monomeric oil after hydrogenolysis of extractives-free white pine sawdust, boron lignin, FA lignin, and Klason lignin generated from both white pine and beechwood are reported in Figures **S3.10-S3.11**.

S1.2.6 Silylation of boron lignin

The procedure was conducted according to literature.[3] Boron lignin (60.0 mg) was transferred to a 100 mL RBF containing a stir bar. BSTFA (60.0 mL) was added. The reaction mixture was heated to 80 °C and stirred vigorously for 1 h. The mass of resultant silylated boron lignin was 86.0 mg (60.0 mg insoluble, 26.0 mg soluble).

Subsequent ^{1}H NMR analysis of the solubilized portion showed a complete absence of aromatic signals, which are present in lignin and lignin derivatives.

S1.2.7 Acetylation of boron lignin

The procedure was conducted in accordance to literature.[4] Boron lignin (51.0 mg) was dissolved in pyridine (0.204 mL) in a 5 mL RBF. Acetic anhydride (10.0 equivalents) was added, and the reaction mixture was stirred at room temperature for 18 h. HCl (1%, 10.0 volumes) was added at 0 °C, and the resulting precipitate was filtered and washed with water to neutral pH. The acetylated lignin was dried in an oven (40 °C) overnight. The mass of resultant acetylated boron lignin was 41.0 mg (33.0 mg insoluble, 8.00 mg soluble).

Subsequent ^{1}H NMR analysis of the solubilized portion showed a complete absence of aromatic signals, which are present in lignin and lignin derivatives.

S1.2.8 Boron lignin solubility test

Boron lignin (54.0 mg) was dissolved in DMSO (3.00 mL) and sonicated for 2 days at 40 °C. The remaining solid was filtered through a glass filter paper under vacuum. The filtrate was concentrated via bulb-to-bulb transfer to obtain a dried residue (9.00 mg). The remaining solid residue was oven dried (100 °C) overnight (42.0 mg). The procedure was repeated with DMSO-d_6 (3.00 mL), and the filtrate was not concentrated down.

Subsequent ^{1}H NMR analysis of the solubilized portion showed a complete absence of aromatic signals, which are present in lignin and lignin derivatives.

S1.3 Analytical Methods

(S1.3.1) Lignin monomer analysis by GC-FID and GC-MS

To analyze lignin monomers after hydrogenolysis, an aliquot (1.00 mL) of the resultant solution (oil dissolved in 5.00 mL or 25.0 mL CH_2Cl_2) was transferred to an autosampler vial with the addition of 50.0 μL of prepared internal standard (20.9 mg decane in 5.00 mL 1,4-dioxane). The solution (~1.05 mL) was analyzed with a Shimadzu GC-2010 Pro equipped with a column (25.0 m length, 0.320 mm inner diameter) and a flame ionization detector (FID). The injection temperature was 300 °C. The column temperature program was: 50 °C (1 min), 15 °C/min to 300 °C (22 min), and 300 °C (7 min). The detection temperature was 300 °C. For the compounds for which commercial standards were not acquired, sensitivity factors of the products were obtained using estimates based on the effective carbon number (ECN).[5] Yield calculations and validation experiments for this method are detailed in Section S2.1.

Monomer peaks for which standards were not available were identified by GC-MS using an Agilent 7250 GC/Q-TOF with an 8890 GC equipped with a 30 m HP-5 ms column and 20:1 split ratio. The peaks in the GC-MS chromatogram appear in the same order as those in GC-FID chromatogram due to the use of a similar capillary column. The conditions used were as follows: a column temperature program of 50 °C (2 min), 10 °C/min to 300 °C (27 min), 300 °C (1 min), and a helium flow of 1 mL/min.

S1.3.2 Gel NMR (2D HSQC) experiment of extracted lignin

This procedure is a modification of a reported protocol.[6] To a 5 mm NMR tube was added boronlignin or FA lignin (30.0-60.0 mg). The lignin was evenly dispersed along the length of the horizontally positioned NMR tube. A 4:1 mixture DMSO-d_6/pyridine-d_5 was transferred into the NMR tube on the bottom and along the sides. The NMR tube was vortexed (700 RPM) using a digital vortex mixer (Fisher Scientific) for 5 min and sonicated for 2 days to make the mixture as homogenous as possible. 2D HSQC spectra of these samples were acquired using a Bruker NEO-500 spectrometer, equipped with a 5 mm probe (PABBO BB-^{1}H/D Z-GRD). The NEO-500 spectrometer operated at a ^{1}H frequency of 500 MHz. The temperature of the samples was controlled by a gas stream passing over the sample, heated as necessary to achieve the desired temperature (300 K, typically). The chemical shift was set via the solvent resonance to correspond to tetramethylsilane (TMS) = 0 parts per million (ppm) (e.g. $CDCl_3$ was referenced as 7.26 ppm).

The 2D parameters used are as follows:

	F1 (H1)	F2 (C-13)
SW	11.8	220
O1p	5.3	100
TD	4k	1k
D1 (relaxation delay)	0.75	
AqTime	0.36	
SI	32k	4k
Weighting Function	gaussian	Qsine
Weighting Factor	-0.5 / 0	SSB = 2

The experiment was conducted in 72 scans with a total acquisition time of 42 h.

S1.3.3 2D HSQC experiment of the hydrogenated oils from extractives-free beechwood sawdust and from boron lignin generated from beechwood

The sample was dissolved in $CDCl_3$ (0.600 mL) and transferred to a 5 mm NMR tube. 2D HSQC spectra were acquired using a Bruker NEO-400 spectrometer, equipped with a prodigy cryoprobe (CPP1.1 BBO 40051 BB-H&F D-05ZxT). The chemical shift was set via the solvent resonance to correspond to TMS = 0 ppm (e.g. $CDCl_3$ was referenced as 7.26 ppm).

The 2D parameters used are as follows:

	F1 (H1)	F2 (C-13)
SW	11.05	219.9
O1p	5	100
TD	16k	256
D1 (relaxation delay)	0.5	
AqTime	1.85	0.0058
SI	32k	1024
Weighting Function	Qsine	Qsine
Weighting Factor	2	2

The experiment was conducted in 32 scans with a total acquisition time of 5.5 h.

S1.3.4 Solid-state NMR spectroscopy of extracted lignin (white pine) and extractives-free white pine sawdust

Solid-state nuclear magnetic resonance (NMR) experiments were performed in a 400 MHz wide bore Bruker ASCEND magnet with a Bruker AVANCE NEO spectrometer running on TopSpin 4.1.4. Samples were packed into a 4 mm rotor and spun at 8,000 Hz at the magic angle in a Bruker H/X double resonance 4 mm magic angle spinning probe. For the multiple-contact cross

polarization (CP) experiments, the recovery delays between the 7 repetitions of the CP step were set to $5x^1HT_1$ (where the $^1HT_1$ is the longitudinal relaxation time determined for each sample by saturation recovery experiments). The ^1H $\pi/2$ pulse was determined to be 3.1 μs at 80 W radiofrequency power, while the ^{13}C $\pi/2$ pulse was determined to be 3.5 μs at 80 W radiofrequency power. The ^1H contact pulse was ramped 50% during the CP transfer step, and tppm15 ^1H decoupling was used during signal acquisition.

S1.3.5 Carbohydrate analysis of boron lignin (white pine, beechwood), FA lignin (white pine, beechwood), and extractives-free sawdust (white pine, beechwood)

Dried lignin samples (~300. mg) were hydrolyzed with 3.00 mL 72 wt% H_2SO_4 for 1 h at 30 °C. The hydrolysate was then diluted to 4 wt% with water. The mixture was then autoclaved at 121 °C for 1 h. The hydrolysis solution was cooled to room temperature and filtered. The filtrate was neutralized pH to ~5.7 and filtered with a 0.22 μm-syringe filter for further HPLC analysis.

High performance liquid chromatography (HPLC) analysis was performed in a Waters 1515 system with a 2414 RI detector on a two-column sequence of Biorad Aminex HPX-87P column (300 x 7.80 mm). Water was used as eluent, and the flow rate was 0.6 mL/min. The injection volume was 20 μL. External calibration curves were constructed by six sugar samples (glucose, xylose, galactose, arabinose, and mannose) at 5 different concentrations. The amount of carbohydrates was calculated based on these calibration curves.

S2. Supplementary Text

S2.1 Monomer Identification and Quantification

S2.1.1 Monomer identification

Initial monomer identification was performed by GC-MS. The GC-MS spectra of identified monomers from the boron lignin are shown in **Figure S3.12**. A GC chromatogram from a white pine and beechwood-derived sample is shown with the identified lignin monomers in **Figure S3.13**.

Whenever possible, the GC-MS retention times of the lignin samples were compared to those of a monomer mixture with authenticated standards. Authentic standards were available from commercial sources for 2-methoxy-4-propylphenol, 2-methoxyphenol, 1,2-dimethoxy-4-propenylbenzene, 3-hydroxy-4-methoxybenzoic acid, 4-hydroxy-3-methoxybenzenepropanoic acid, 1,2-dimethoxy-4-propylbenzene, 2-methoxy-4-methylphenol, 2-methoxy-4-ethylphenol, 2,6-dimethoxy-4-ethylphenol, 2,6-dimethoxy-4-methylphenol, and 2,6-dimethoxy-4-propylphenol. The characterization of these lignin monomer standards and their comparison with those identified in the lignin monomer mixture are shown in **Figure S3.14**. For the monomers for which authenticated standards were unavailable, their identification was predicted based on their m/z and fragmentation patterns. The characterization of these lignin monomers is shown in **Figure S3.15**.

S2.1.2 Monomer quantification and yield calculations

Calculations of monomer yield based on lignin-rich solid were conducted as previously reported,[2] and additional calculations of monomer yield based on hydrogenated oil were also performed. Since a large quantity of monomers with similar masses and connectivity were produced, quantification was based on an internal standard (decane) and calibration by relative response factors (RRFs) of commercial standards or the ECN rule for compounds identified without commercial standards. As stated in Section **S1.3**, 50.0 μL of an internal standard solution (20.9 mg decane in 5.00 mL 1,4-dioxane) was added to 1.00 mL of the lignin monomer solution to be analyzed. The resulting solution (~1.05 mL) was analyzed by GC-FID. Monomer yields were calculated based on their areas and the area of decane in the GC-FID chromatogram. The detailed calculations are as follows:

$$n_{decane} = \frac{W_{decane\ in\ sample}}{MW_{decane}} \quad \text{(S1)}$$

$$n_{monomer} = \frac{A_{monomer\ in\ sample}}{A_{decane\ in\ sample}} \times n_{decane} \times \frac{ECN_{decane}}{RRF_{monomer}} \quad \text{(S2)}$$

$$W_{monomer} = n_{monomer} \times MW_{monomer} \quad \text{(S3)}$$

$$Y_{monomer} = \frac{W_{monomer} \times V}{W_{Klason\ lignin}} \times 100\% \quad \text{(S4)}$$

The variables in the equations are as follows:

$W_{decane\ in\ sample}$ (mg): the weight of decane used as an internal standard in each GC sample

MW_{decane} (mg/mmol): the molecular weight of decane (142 mg/mmol)

n_{decane} (mmol): the molar amount of decane in each GC sample

$n_{monomer}$ (mmol): the molar amount of monomer in each GC sample

$A_{monomer\ in\ sample}$: the peak area of monomer in the GC-FID chromatogram

ECN_{decane}: the effective carbon number (10) of decane

$RRF_{monomer}$: the relative response factor of the lignin monomer molecule

$W_{monomer}$ (mg): the molecular weight of a β–O–4-bonded monomer guaiacyl glycerol (196 mg/mmol) or a β–O–4-bonded syringyl glycerol (226 mg/mmol) or a β–O–4-bonded *p*-hydroxyphenyl unit in the GC sample; known molecular weights of authenticated standards were used in the calculation

$W_{Klason\ lignin}$ (mg): the amount of native lignin in the extracted lignin-rich solid; the Klason protocol was performed on boron-lignin and FA lignin to determine this

$Y_{monomer}$: the yield of monomer based on the weight of extracted lignin

V (mL): the total volume of sample, 1 mL of which was used for GC analysis

Monomer yield in the hydrogenated oil was also calculated for each sample. The detailed calculations are as follows:

$$Y_{monomer} = \frac{n_{monomer} \times MW_{standard} \times V}{W_{oil}} \times 100\% \qquad \text{(S5)}$$

The variables in the equation are as follows:

$MW_{standard}$ (g/mol): the molecular weight of the authenticated standard

W_{oil} (mg): the measured weight of the oil after hydrogenolysis.

S2.1.3 Validation of monomer quantification

The ECN rule is a commonly used method to quantify carbon-containing molecules based on their predicted GC-FID RRFs. In the case where authenticated standards of monomers were available, the RRF was calculated as shown in equation S6, and used in equation S2:

$$RRF_{monomer} = \left[\frac{A_{monomer}}{A_{decane}} \times \frac{n_{decane}}{n_{monomer}}\right] \times 10 \qquad \text{(S6)}$$

The ECN can also be calculated theoretically as previously described, which was done with monomers for which authenticated standards were unavailable.[2] All predicted ECNs and measured RRFs of lignin monomers (ECN or $RRF_{monomers}$) used for quantification are summarized in **Table S4.3**.

S3. Figures

	Extractives-free sawdust	BBr_3 (8 mmol, 1 M CH_2Cl_2)<br>BCl_3 (8 mmol, 1 M CH_2Cl_2)<br>→<br>CH_2Cl_2, rt<br>18h	Lignin-rich solid residue	organic layer
White pine	500. mg		Treatment 1: 371 mg	5.00 mg
			Treatment 2: 300. mg	4.00 mg
			Treatment 3: 221 mg	5.00 mg
			Treatment 4: 169 mg	5.00 mg
			Treatment 5: 136 mg	6.00 mg
Beechwood	500. mg		Treatment 1: 309 mg	9.00 mg
			Treatment 2: 239 mg	7.00 mg
			Treatment 3: 168 mg	5.00 mg
			Treatment 4: 122 mg	3.00 mg

Figure S3.1. Boron lignin extraction protocol for white pine and beechwood. The sugar extraction from lignocellulose results in a lignin-rich solid residue. The mass of the residue and organic layer are shown after each boron trihalide treatment.

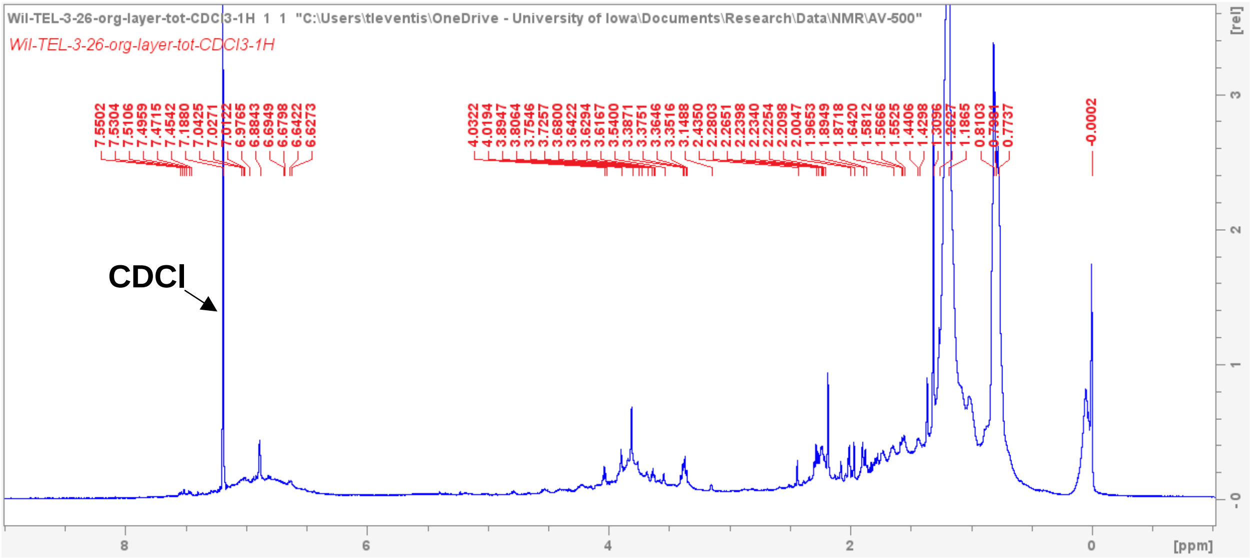

Figure S3.2. ^{1}H NMR of the combined organic layers after boron lignin separation protocol (white pine). The ^{1}H NMR of the total organic layer in $CDCl_3$ after five treatments of mixed boron trihalides is shown. The mass of the organic layers combined resulted in 5.00 wt% (25.0 mg) of the lignocellulosic biomass (500. mg).

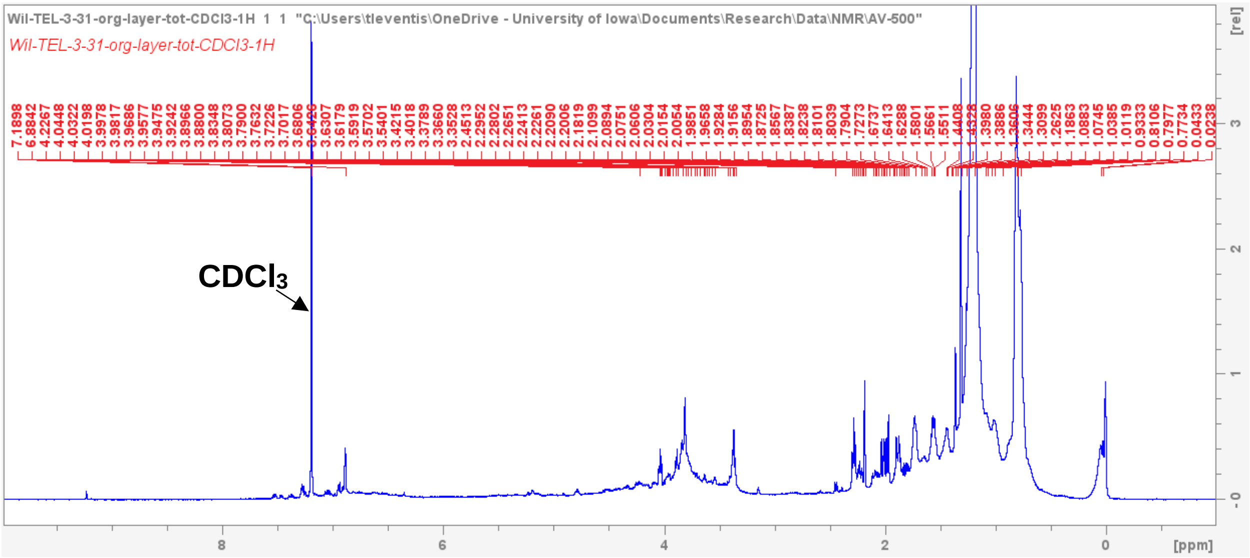

Figure S3.3. ¹H NMR of the combined organic layers after boron lignin separation protocol (beechwood). The ¹H NMR of the total organic layer in $CDCl_3$ after four treatments of mixed boron trihalides is shown. The mass of the organic layers combined resulted in 4.80 wt% (24.0 mg) of the lignocellulosic biomass (500. mg).

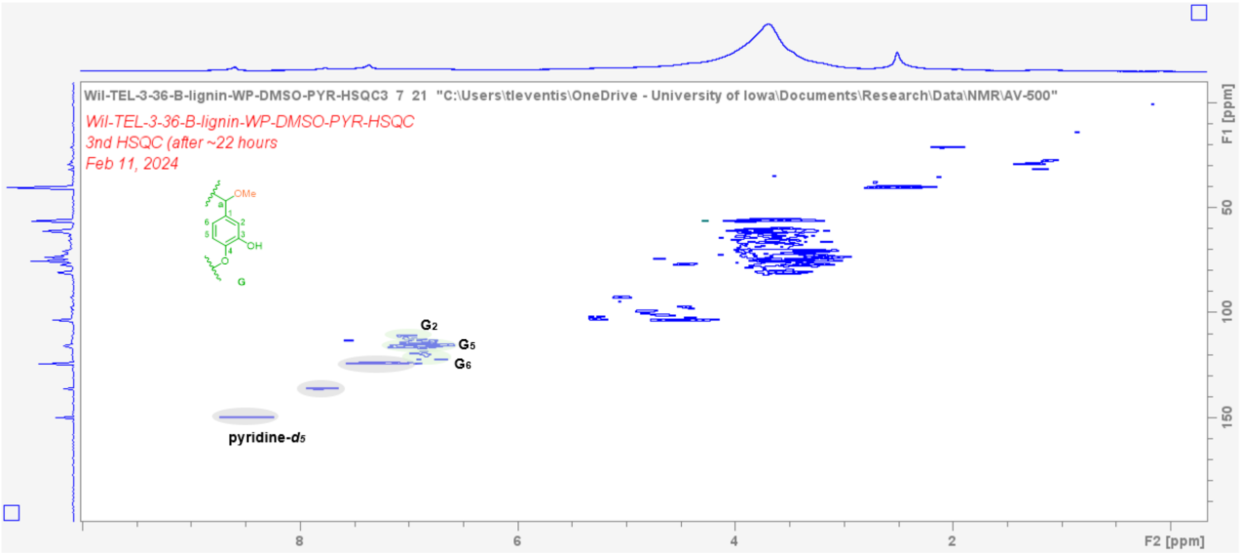

Figure S3.4. Gel 2D HSQC of boron lignin generated from extractives-free white pine sawdust. The sample was prepared by mixing the lignin-rich solid (60.0 mg) with 4:1 DMSO-d_6/ pyridine-d_5 in a 5 mm NMR tube and sonicating until the sample appeared homogenous (5 h). By the end of the experiment, the gel settled to the bottom of the NMR tube. The spectrum shows protons attributed to guaiacyl units as expected for white pine, a softwood.[2]

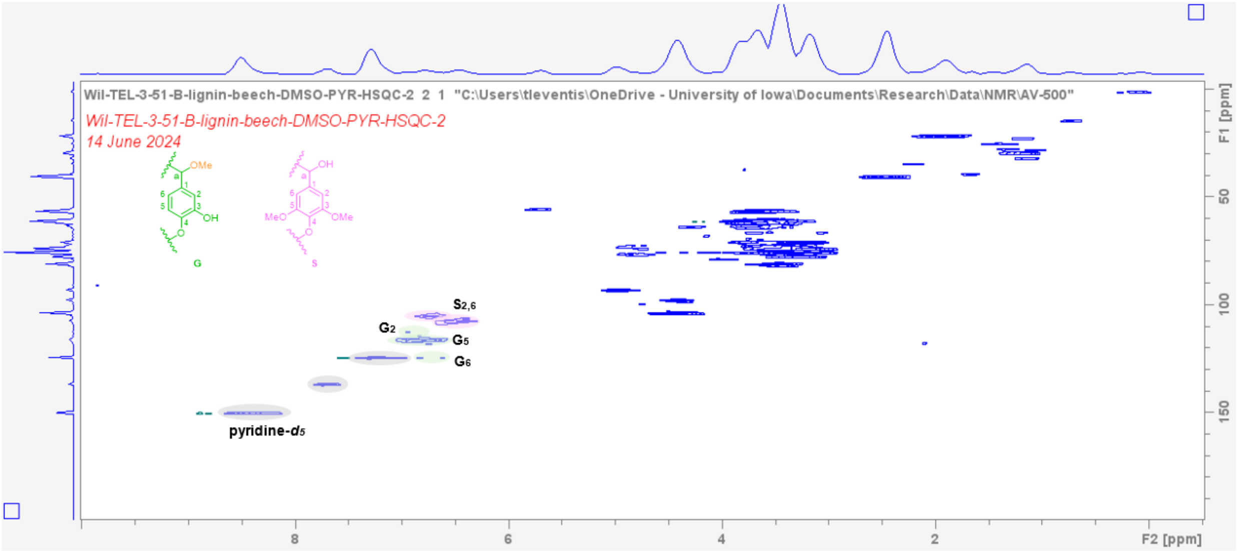

Figure S3.5. Gel 2D HSQC of boron lignin generated from extractives-free beechwood sawdust. The sample was prepared by mixing the lignin-rich solid (60.0 mg) with 4:1 DMSO-d_6/ pyridine-d_5 in a 5 mm NMR tube and sonicating until the sample appeared homogenous (5 h). The spectrum shows protons attributed to guaiacyl and syringyl units as expected for beechwood, a hardwood.[2]

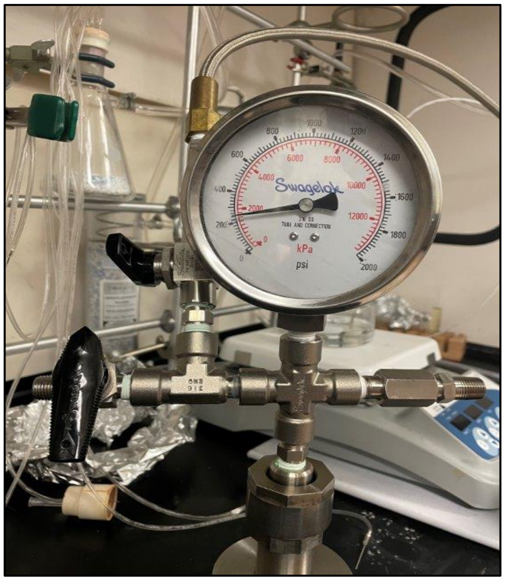

50 mL Parr Bomb with H_2 gas inlet

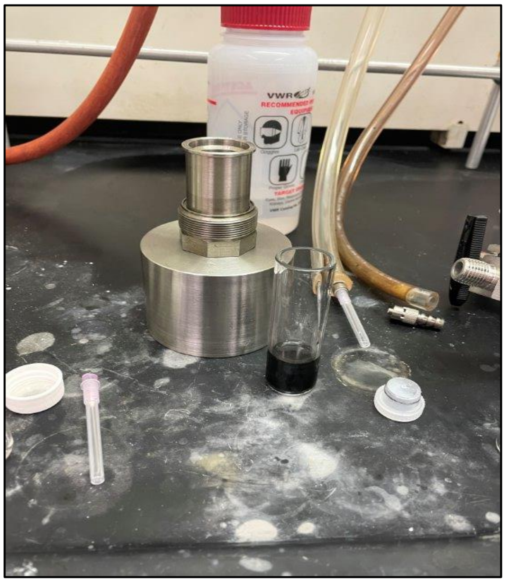

25 mL glass insert containing reaction mixture

Figure S3.6. Custom-made Parr bomb (50 mL) with H_2 gas inlet (left) and custom-made glass insert (25 mL, right). The glass insert in the image contains the reaction mixture at the start of reaction.

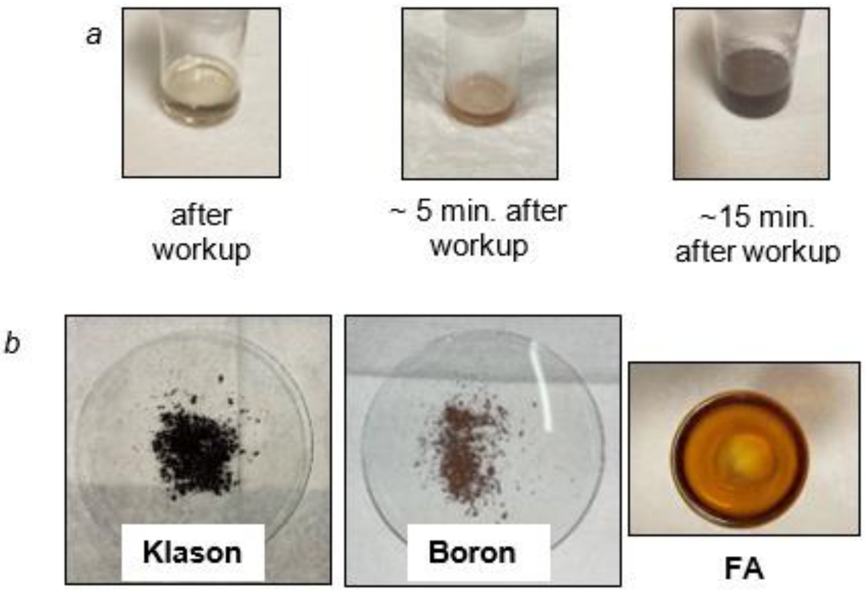

Figure S3.7. [a]Progressive monomer degradation of the beechwood monomer oil upon exposure to air. Longer exposure time resulted in a strong color change from a pale yellow after workup to a light pink 5 min after workup to a dark brown 15 min after workup. The color change was accompanied with the formation of insoluble particulates. [b]Comparison of color for high-condensed lignin (Klason lignin, white pine) and low-condensed lignin (boron lignin and FA lignin, white pine).

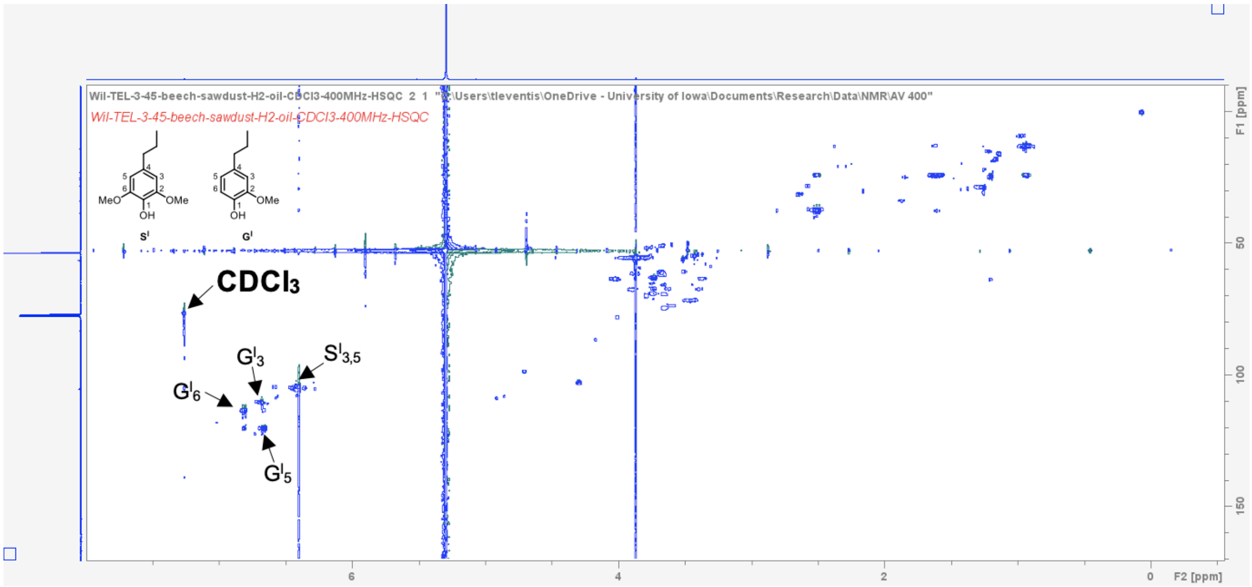

Figure S3.8. 2D HSQC of the hydrogenated oil of the extractives-free beechwood sawdust sample.

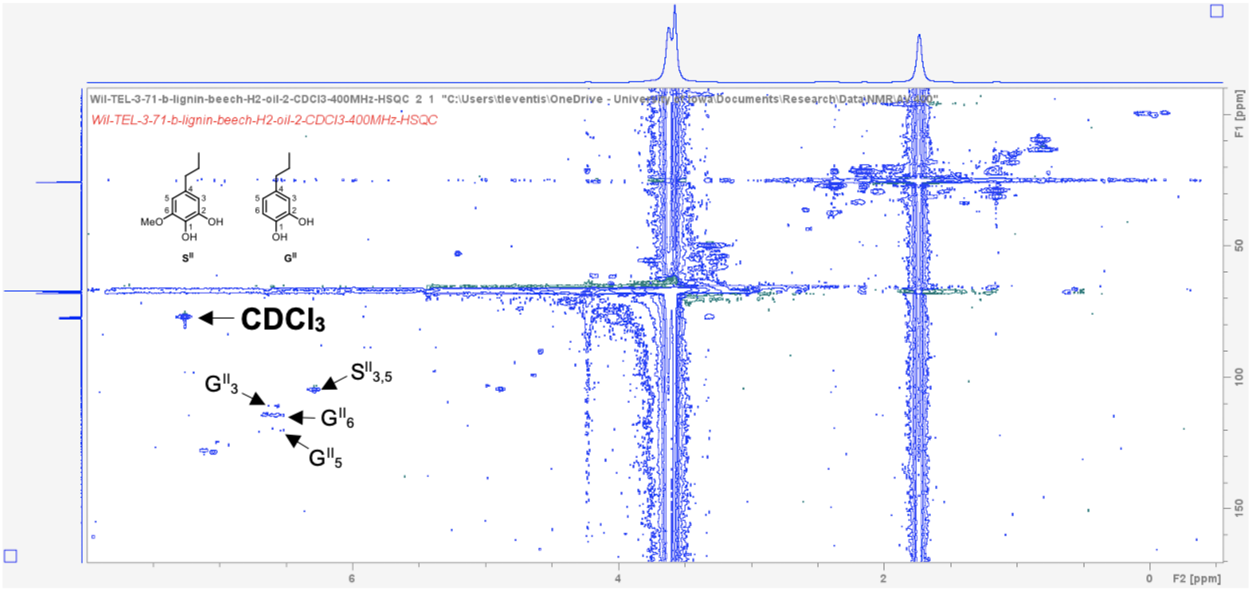

Figure S3.9. 2D HSQC of the hydrogenated oil of the boron lignin sample generated from extractives-free beechwood sawdust.

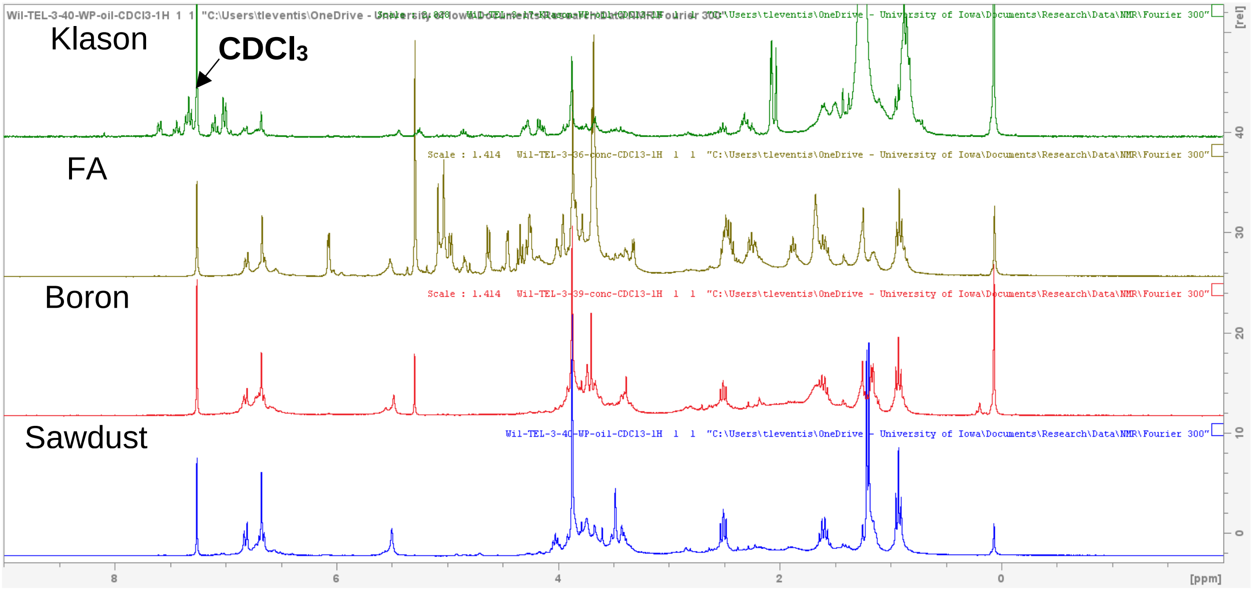

Figure S3.10. Comparison of ^{1}H NMRs of hydrogenated oils from extractives-free white pine sawdust, boron lignin, FA lignin, and Klason lignin. The spectra were acquired using a Bruker NEO-300 spectrometer.

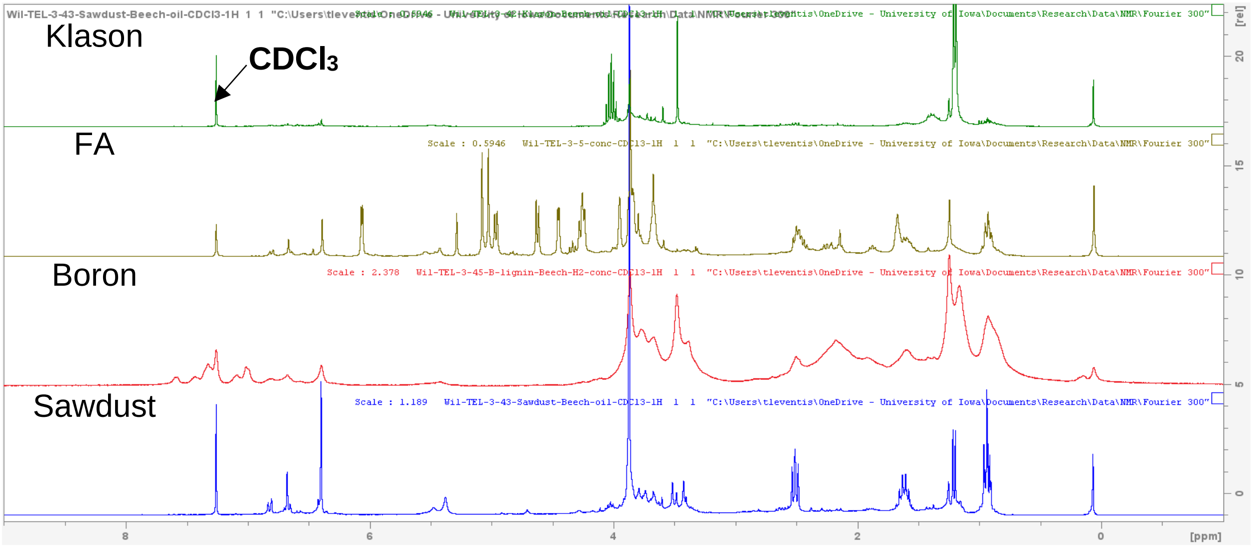

Figure S3.11. Comparison of ^{1}H NMRs of hydrogenated oils from extractives-free beechwood sawdust, boron lignin, FA lignin, and Klason lignin. The spectra were acquired using a Bruker NEO-300 spectrometer.

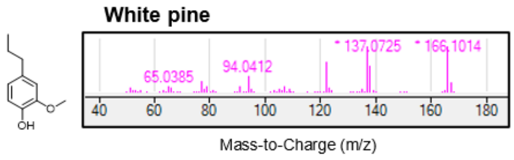

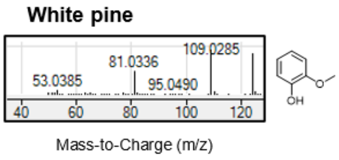

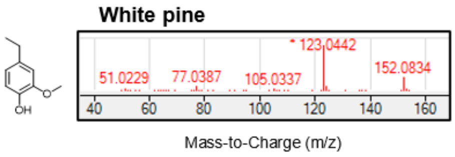

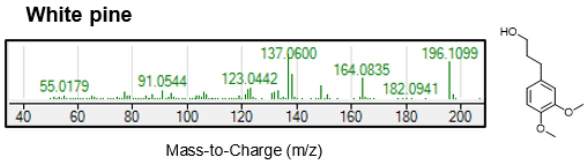

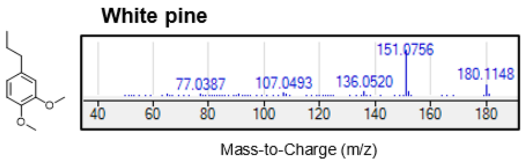

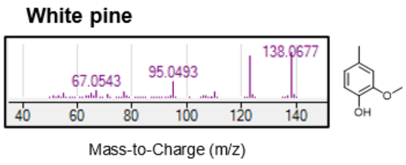

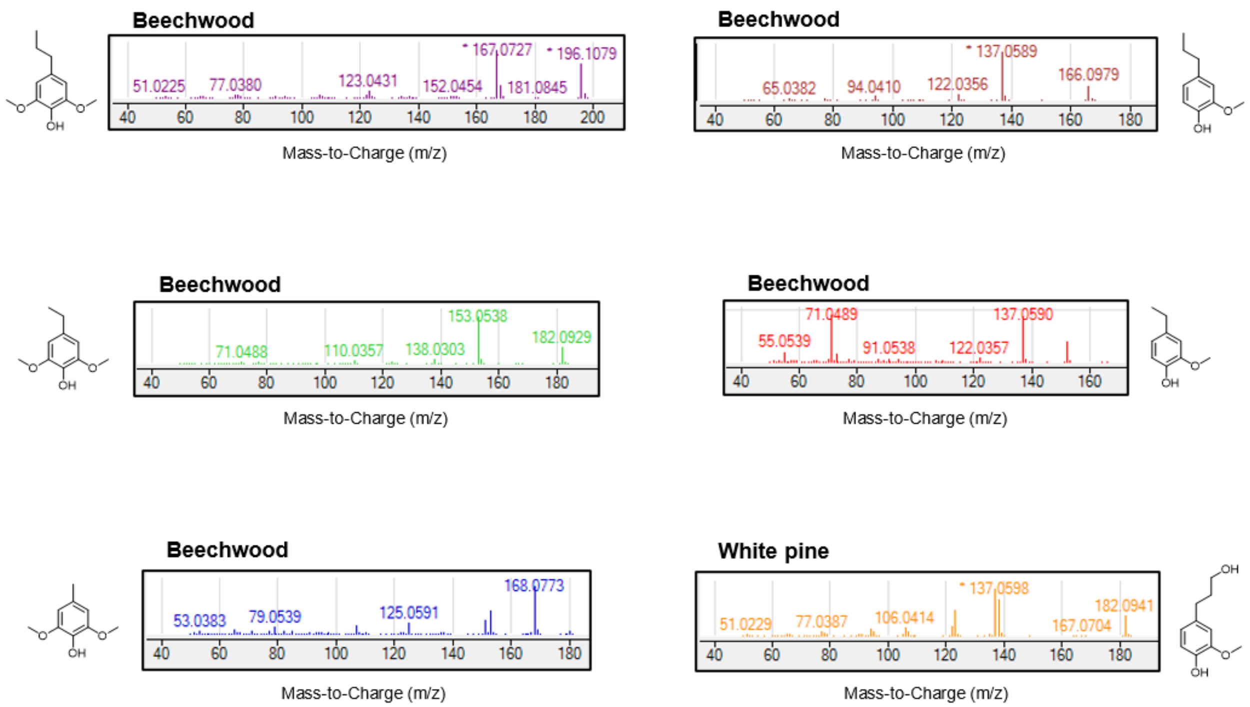

Figure S3.12. GC-MS spectra of monomeric oil resulting from the hydrogenolysis of boron lignin generated from white pine or beechwood sawdust. The mass, fragmentation pattern, and retention time of the monomers match those of authentic standards.

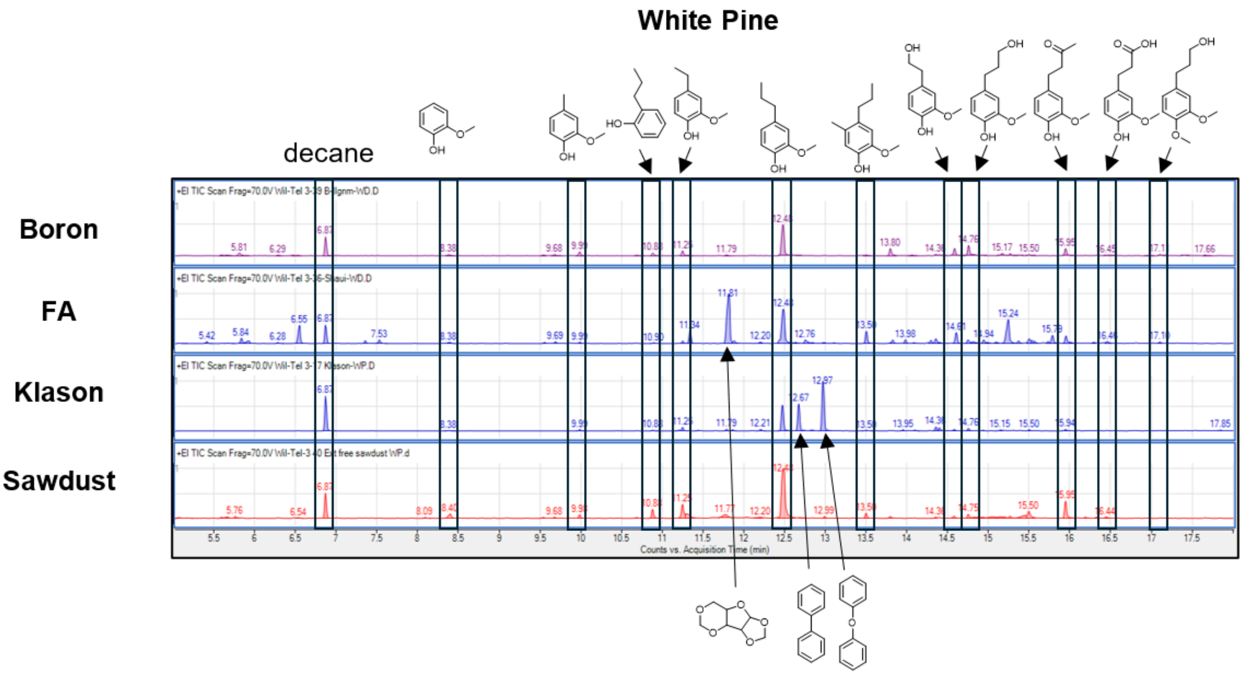

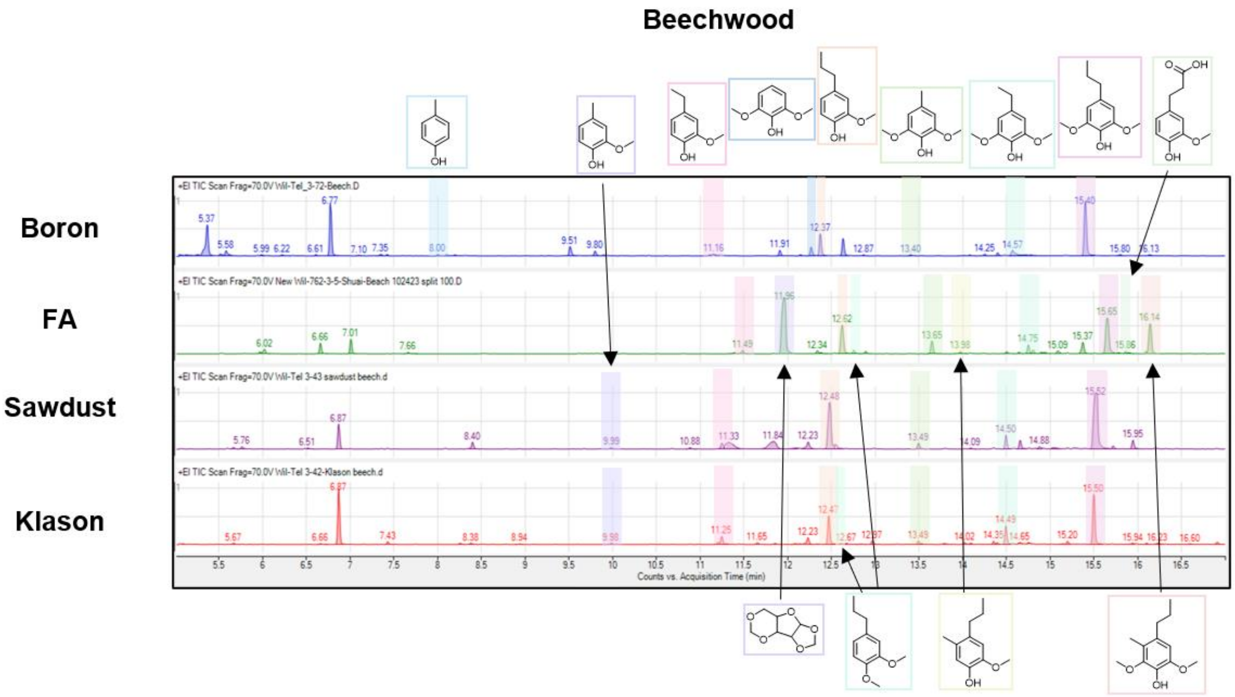

Figure S3.13. Comparison of the GC-MS chromatogram of boron lignin, FA lignin, Klason lignin, and extractives-free sawdust monomer mixtures of a white pine and beechwood-derived sample with the identified lignin monomers. Note: For the white pine-derived chromatograms, the peak attributed to the methylated propylguaiacol (13.5 min) in the FA lignin chromatogram is *not* present in the boron lignin or sawdust chromatograms. For the beechwood-derived chromatograms, the samples were run at two different time points. The column was refreshed in between, causing the peak shift. Authenticated standards were run at those timepoints before and after to be certain of assignments.

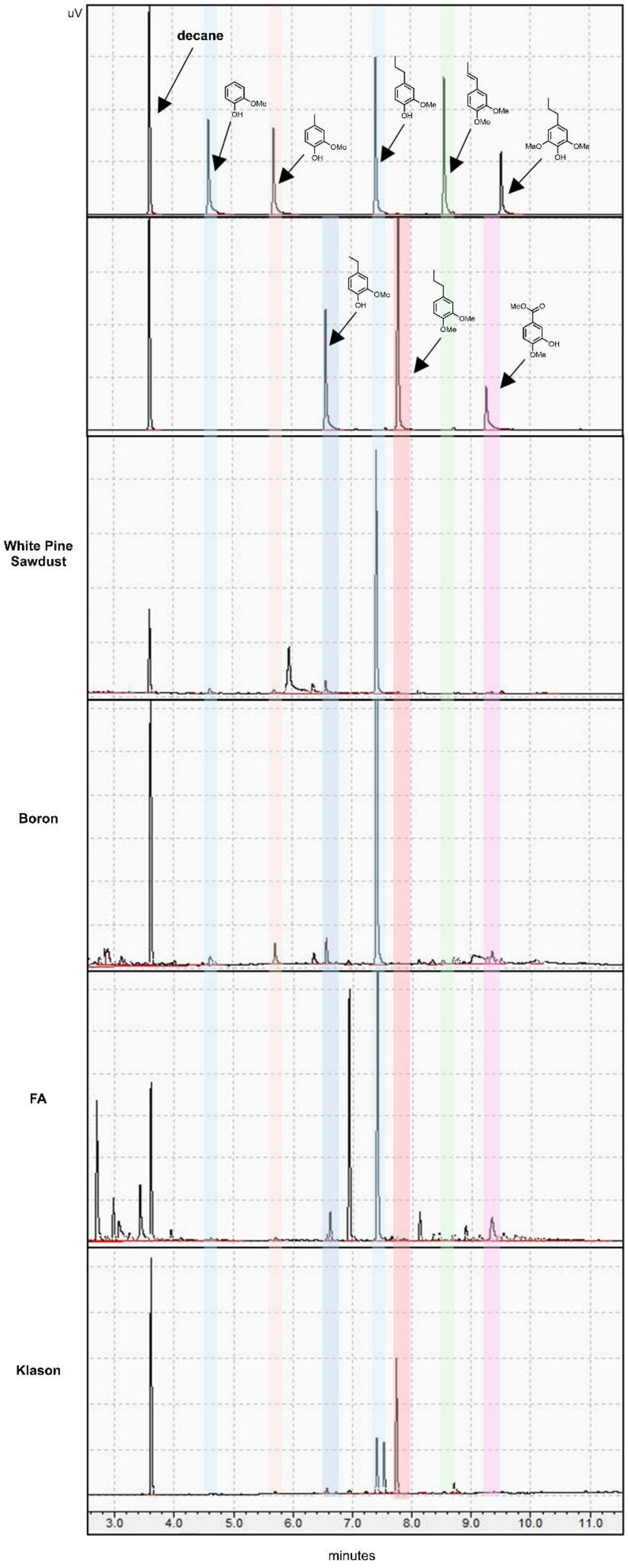

uV
decane
OMe
OH
White Pine Sawdust
Boron
FA
Klason
3.0
4.0
5.0
6.0
7.0
8.0
9.0
10.0
11.0
minutes

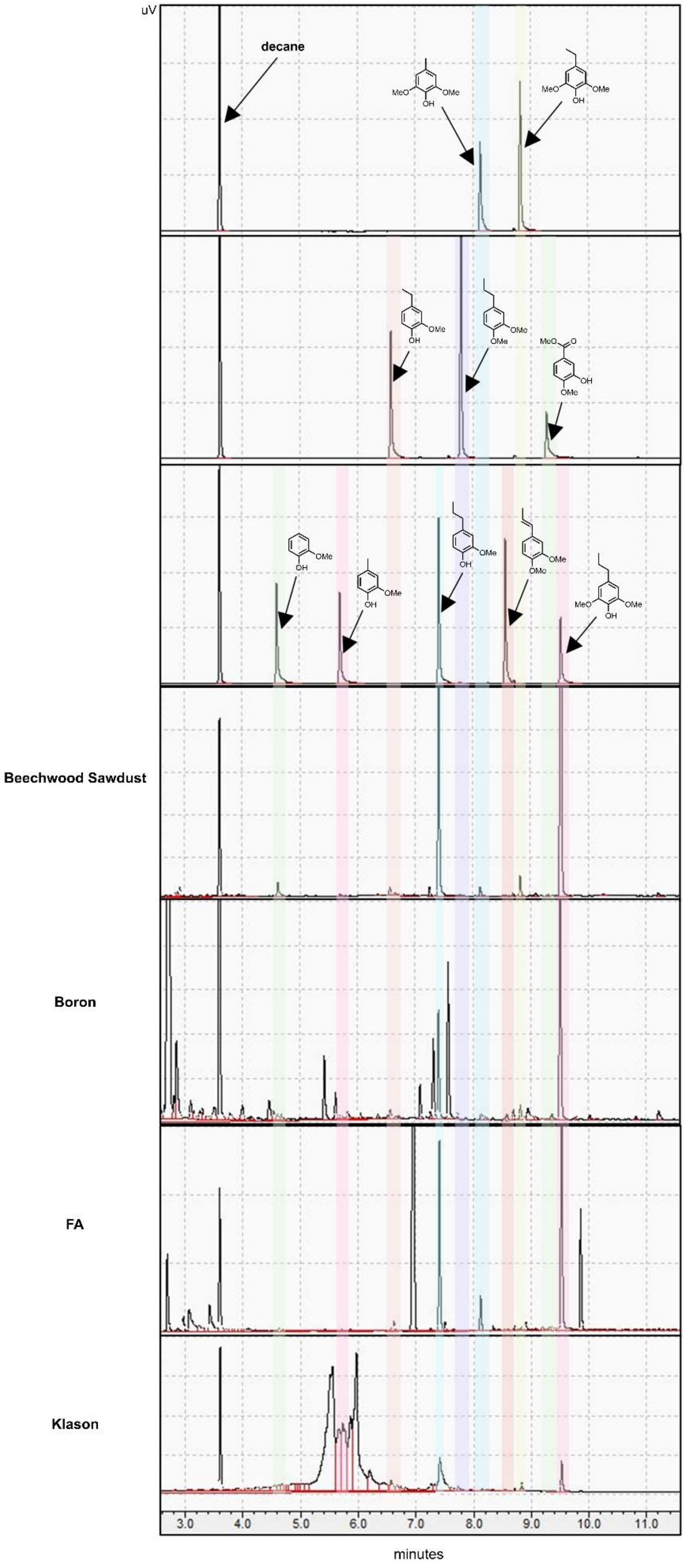
uV
decane
MeO
OMe
OH
OMe
MeO
Beechwood Sawdust
Boron
FA
Klason
3.0
4.0
5.0
6.0
7.0
8.0
9.0
10.0
11.0
minutes

Figure S3.14. Comparison of the GC-FID chromatograms of the boron lignin, Klason lignin, and FA lignin monomer mixtures (generated from white pine – S18 - and beechwood – S19) showing the identified monomers, and the standard monomer mixture. *Note:* “uV” was generated by the GC-FID and does *not* represent absorption.

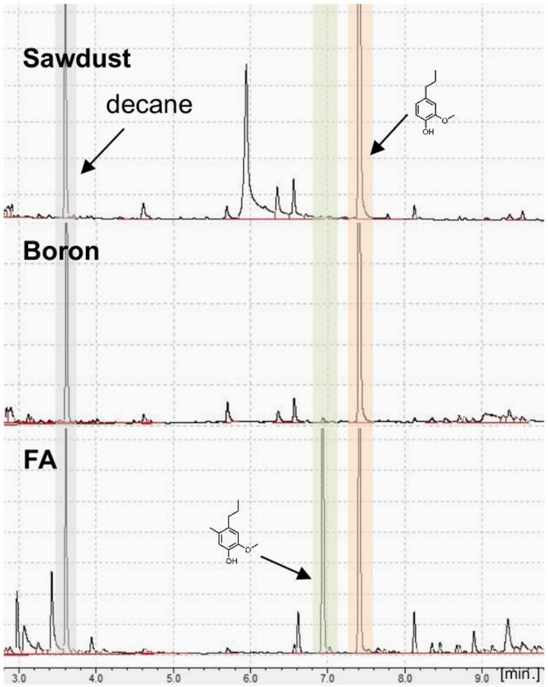

Figure S3.15. Comparison of a zoomed in window of the GC-FID chromatogram of extractives-free sawdust, boron lignin, Klason lignin, and FA lignin (generated from white pine) monomer mixtures.

Beechwood

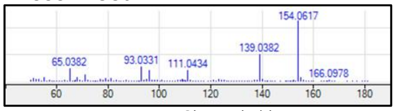

Mass-to-Charge (m/z)

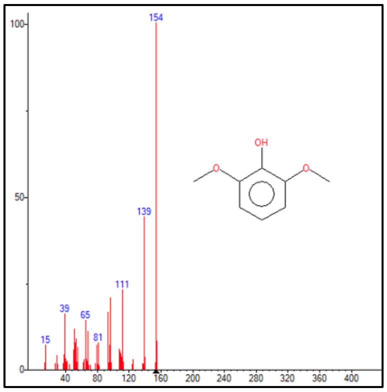

White Pine

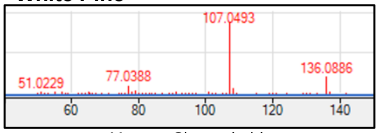

Mass-to-Charge (m/z)

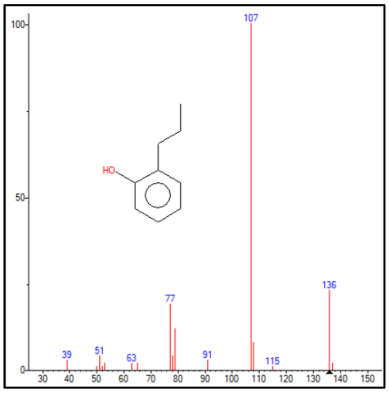

White Pine

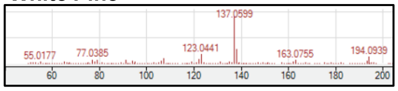

Mass-to-Charge (m/z)

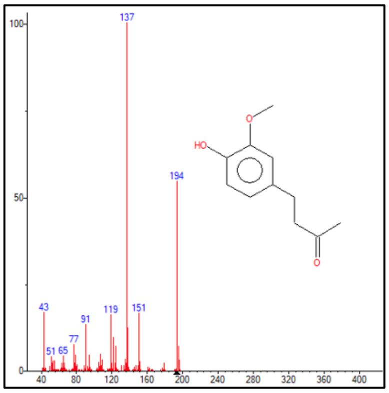

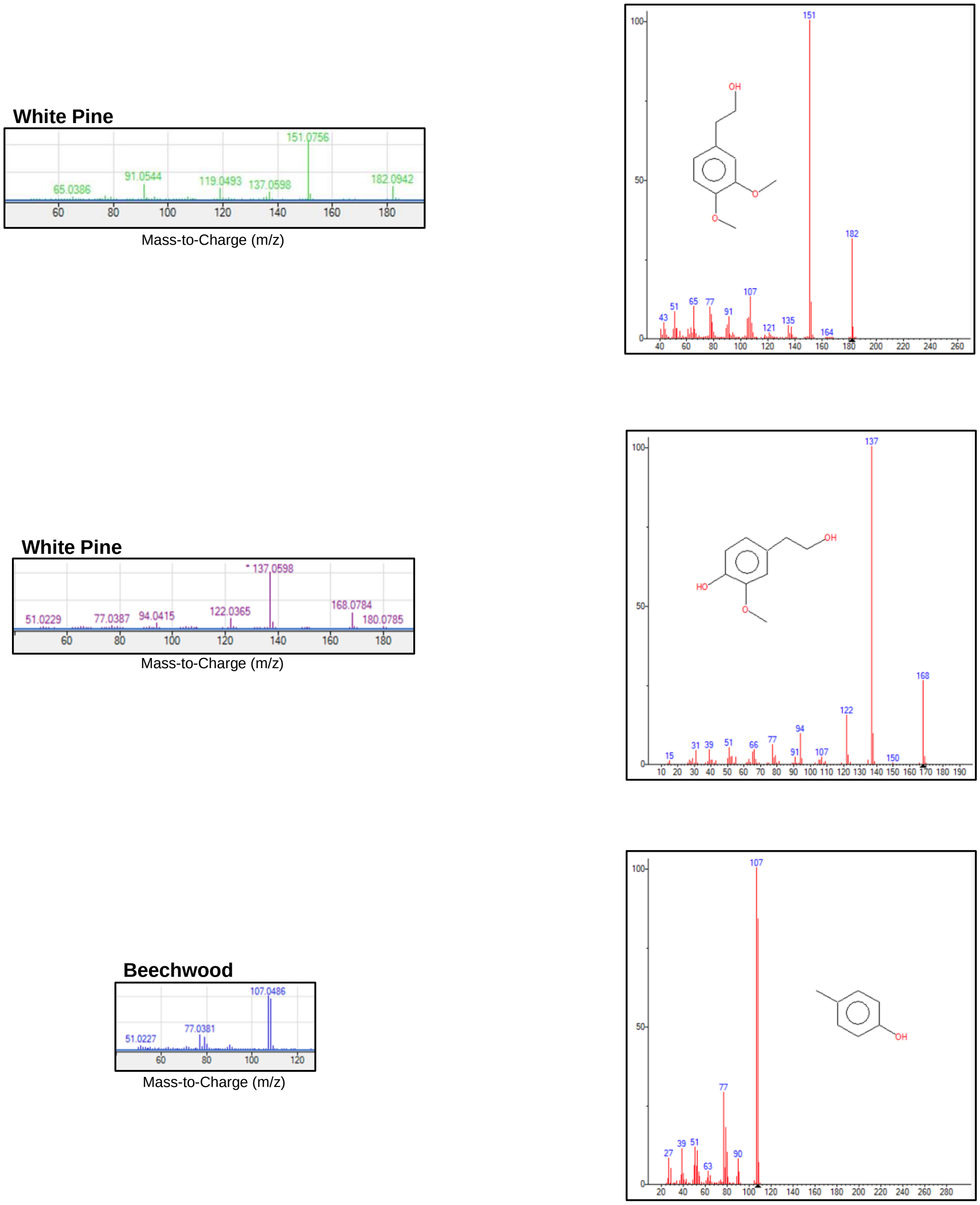

Figure S3.16. GC-MS of boron lignin monomers for which authenticated standards were not obtained and their respective MS library predictions (S21-22). M^+ is the molecular ion peak and represents the molecular weight of lignin monomers. The highest peak in the spectra represents the molecular weight of the most stable ion fragment from each lignin monomer.

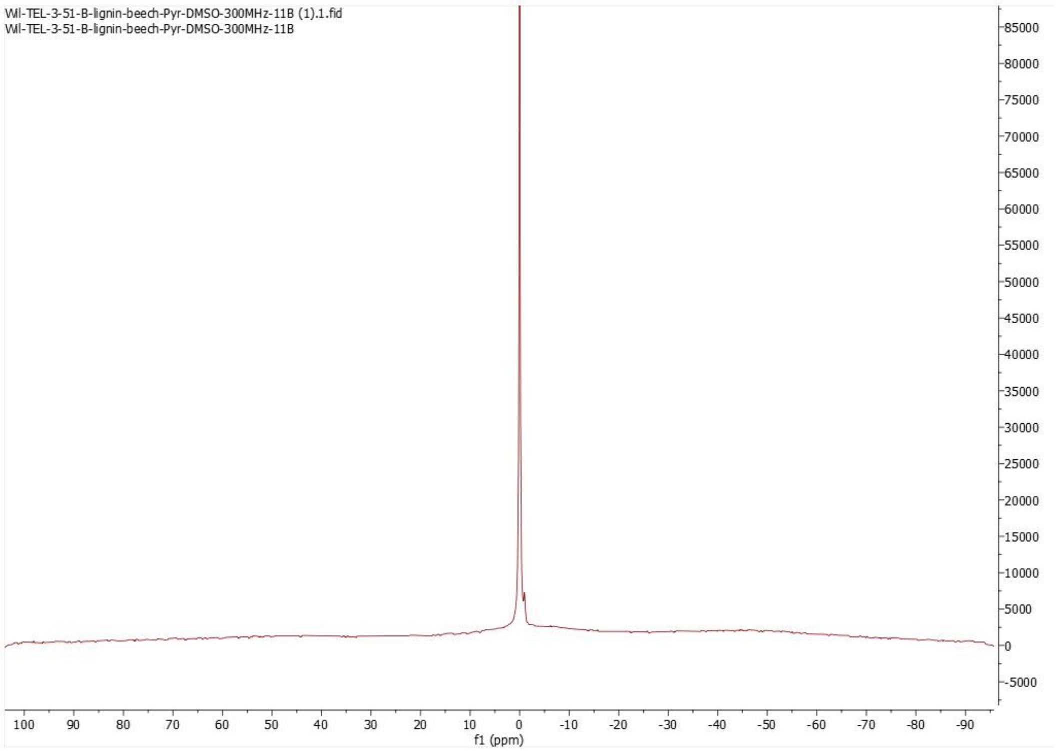

Figure S3.17. ^{11}B NMR of boron-lignin (derived from beechwood). $BF_3O(CH_2H_5)_2$ was used as an internal standard. There is a complete absence of other boron peaks present, suggesting that the lignin sample is not coordinating to any boron species.

Tables

Table S4.1. Summary of lignin-rich mass extracted, and the amount of remaining sugar extracted from lignin-rich mass following the Klason protocol. [a]Originating from white pine sawdust. [b]Originating from beechwood sawdust. Individual sugar monomer content for boron lignin samples was assessed based on a 372 mg scale for white pine and 150 mg scale for beechwood. NA = not assessed. ND = not detected. Some sugar monomers for boron lignin samples were not assessed due to unknown background signal complicating the assessment of those sugars.

Entry	Method	Sawdust (mg)	Lignin-rich mass (mg)	Carbohydrates remaining (wt %)	Glucose (wt %)	Xylose (wt %)	Galactose (wt %)	Arabinose (wt %)	Mannose (wt %)
1	Klason	500	136[a], 113[b]	0[a,b]	0[a,b]	0[a,b]	0[a,b]	0[a,b]	0[a,b]
2	FA	1000	821[a], 819[b]	80.0[a], 74.0[b]	NA[a], NA[b]	NA[a], NA[b]	NA[a], NA[b]	NA[a], NA[b]	NA[a], NA[b]
3	Boron	500	136[a], 122[b]	15.0[a], 20.0[b]	2.63[a], 30[b]	1.75[a], NA[b]	NA[a], NA[b]	NA[a], NA[b]	NA[a], NA[b]
4	Biomass	200	NA[a], NA[b]	73.0[a], 74.4[b]	44.0[a], 59.2[b]	0.50[a], 24.7[b]	0.83[a], ND[b]	0.20[a], ND[b]	14.7[a], ND[b]

Table S4.2. Optimization of hydrogenolysis reaction. FA lignin generated from extractives-free white pine sawdust was used for optimization due to short reaction time, large amount of lignin produced, and structural simplicity of the softwood. The reactor was pressurized with H_2 at room temperature and is listed in psi. Reaction time began once the reactor was heated to the desired temperature. Three different heat sources were investigated – heat tape ultimately gave the most consistent results. Monomer yield was calculated based on the theoretical number of cleavable monomers provided by the "lignin-first" strategy (23.3%) and the amount of Klason lignin in the lignin-rich material following the extraction protocol.[2] *Catalyst was added batch-wise. Half of the catalyst was added at the beginning, and the other half was added halfway through the reaction (after 12 h). Optimal (entry 16) was used to perform hydrogenolysis on all other lignin samples.

Entry	H_2 (rt, psi)	Ru/C (wt %)	Solvent	Temp (°C)	Volume (mL)	time (h)	solid (mg)	oil (mg)	Monomer yield (%, solid)
1	340	44	THF	250/oil bath	10	6	61	60	33.0
2	360	40	THF	250/oil bath	5	15	66	53	45.5
3	380	42	THF	250/oil bath	3	15	73	29	33.0
4	380	43	THF	270/heat mantle	5	48	75	39	43.8
5	380	34	MeOH	225/heat mantle	5	24	74	57	48.9
6	80	22	MeOH	205/heat tape	10	15	59	25	24.0
7*	70	19	MeOH	250/heat tape	5	12	75	28	6.0
8	80	43	MeOH	250/heat tape	5	15	71	45	28.3
9	400	43	MeOH	150/heat tape	5	24	74	42	12.4
10	380	31	MeOH	220/heat tape	5	24	78	72	41.6
11	380	34	MeOH	220/heat tape	5	24	63	45	28.3
12	380	55	MeOH	210/heat tape	5	24	72	42	24.9
13	460	32	MeOH	220/heat tape	5	24	47	51	20.2
14	380	41	MeOH	230/heat tape	5	24	62	59	35.2
15*	380	26	MeOH	220/heat tape	5	12	67	49	23.6
**16**	**380**	**40**	**MeOH**	**220/heat tape**	**5**	**24**	**5**	**36**	**58.0**

Table S4.3. Effective carbon number (ECN) or relative response factor (RRF) for lignin monomers used in this study.

Lignin monomer structure	$RRF_{monomer}$ or $ECN_{monomer}$	Lignin monomer structure	$RRF_{monomer}$ or $ECN_{monomer}$
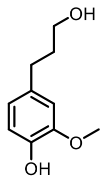	7.40 (RRF)	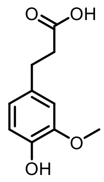	7.40 (ECN)
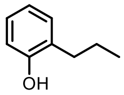	8.00 (ECN)	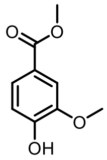	5.22 (RRF)
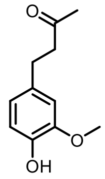	9.00 (ECN)	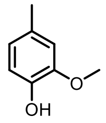	6.12 (RRF)
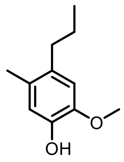	9.00 (ECN)	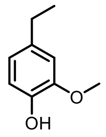	7.10 (RRF)
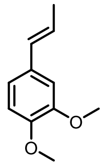	8.55 (RRF)	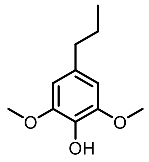	6.74 (RRF)
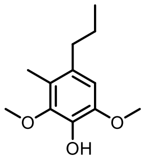	8.40 (ECN)	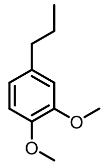	9.61 (RRF)
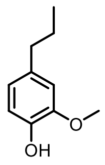	8.47 (RRF)	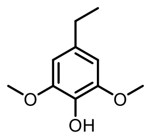	7.24 (RRF)
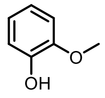	5.25 (RRF)	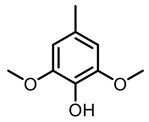	6.09 (RRF)

Table S4.4. Statistics calculations for lignin monomers.
Statistical assessment was done using unpaired student's *t*-test.

White Pine Results

% of Solid

	**Average Yields (%)**				**p values**	
**Monomer**	Sawdust	FA	Boron	Klason	Boron vs. FA	Boron vs. Klason
M1	78.56906	43.45436	34.72959	1.680875	0.241371	0.015949
M2	0	0.983946	0	0	0.018452	
M3	5.557328	0	2.078242	0	0.042878	0.042878
M4	4.261143	0	1.49803	0	0.000726	0.000726
M5	1.126883	2.458311	0.918705	0	0.430037	0.113574
M6	0	3.060029	0	0	0.020056	
M7	0	0.404642	0	0	0.044482	
M8	0	0	1.113976	0	0.038046	0.038046
M9	2.944802	3.009701	3.89433	0.207347	0.587767	0.042545
M10	7.54078	3.009701	2.760783	0.208634	0.863148	0.012625
Diformylxylose	0	22.20233	0	0		
Total	100	56.38069	46.99365	2.096856	**0.2558**	**0.0012**
Standard deviation		7.818699	9.455756	0.327344		

% of Oil

**Average Yields (%)**				**p values**	
**Monomer**	Sawdust	FA	Boron	Boron vs. Sawdust	Boron vs. FA
M1	19.29421	6.705731	9.600638	0.075153	0.040259
M2	0	3.314413	0		0.134402
M3	1.105929	0	0.478767	0.050927	0.048696
M4	0.788636	0	0.314575	0.083268	0.010428
M5	0.325837	0.550432	0.289463	0.780006	0.594944
M6	0	0.507945	0		0.007513
M7	0	0.080284	0		0.119125
M8	0	0	0.33204	0.021866	0.021866
M9	0.593492	0.192036	0.909108	0.244287	0.066996
M10	1.557972	0.199155	0.699909	0.128393	0.007675
Diformylxylose	0	11.35794	0		
Total	23.66608	11.54999	12.6245	**0.0254**	**0.3849**
Standard Deviation	5.242989	0.952247	1.654816		

Beechwood Results

% of Solids

	Average Yields (%)				p values	
**Monomer**	Sawdust	FA	Boron	Klason	Boron vs. FA	Boron vs. Klason
M1	21.72144	13.10268	3.651283	0.891752	0.00367	0.054226
M2	1.143911	0	0.475046	0	0.005197	0.005197
M3	0.417516	0	0.245131	0	0.142393	0.142393
M4	1.762493	0	0.594429	0	0.001615	0.001615
M5	72.38355	39.41814	15.35935	0.835508	0.003205	0.012669
M6	0	6.498032	0	0	0.094372	
M7	0	0.168826	0	0	0.0009	
M8	0	0.322515	0	0.076693	0.096176	0.175638
M9	0.863477	1.498835	0.840737	0	0.524562	0.008078
M10	0	0	0.542828	0	0.000239	0.000239
M11	0	10.30065	0	0	0.000063	
M12	0.763635	0.828893	0	0	0.004633	
M13	0.943981	0.77757	0.580424	0.097233	0.461829	0.002049
Total	100	72.91615	22.28923	1.901185	**0.001**	**0.0092**
Standard Deviation		6.975953	7.325643	1.511706		

% of Oil

	Average Yields (%)			p values	
**Monomer**	Sawdust	FA	Boron	Boron vs. Sawdust	Boron vs. FA
M1	8.128459	4.767568	2.742249	0.003425	0.037726
M2	3.461422	1.470973	0.757377	0.503688	0.070851
M3	0.329717	0	0.153005	0.961663	0.012021
M4	0.455073	0	0.29959	0.166867	0.000628
M5	19.31038	9.551105	8.00921	0.000873	0.571421
M6	10.1114	6.090721	2.232681		0.00033
M7	0	1.317957	0		0.058078
M8	0	0.113609	0		0.002653
M9	0.309033	0.443168	0.488284	0.355796	0.021714
M10	0.028179	0.073459	0.35317	0.125698	0.00074
M11	0	2.675931	0		
M12	0.149794	1.210979	0	0.008628	
M13	0.312407	0.54261	0.205351	0.896602	
Diformylxylose	0.037792	24.67904	0.058783		
Total	42.59586	28.25808	15.24092	**0.0013**	**0.0132**
Standard Deviation	3.237199	2.430322	4.474247		

S4. References

(1) Kazmi, M. Z. H.; Karmakar, A.; Michaelis, V. K.; Williams, F. J. Separation of cellulose/hemicellulose from lignin in white pine sawdust using boron trihalide reagents. *Tetrahedron* **2019**, *75* (11), 1465-1470.

(2) Shuai, L.; Amiri, M. T.; Questell-Santiago, Y. M.; Heroguel, F.; Li, Y.; Kim, H.; Meilan, R.; Chapple, C.; Ralph, J.; Luterbacher, J. S. Formaldehyde stabilization facilitates lignin monomer production during biomass depolymerization. *Science* **2016**, *354* (6310), 329-333.

(3) Morville, S.; Scheyer, A.; Mirabel, P.; Millet, M. A multiresidue method for the analysis of phenols and nitrophenols in the atmosphere. *J. Environ. Monit.* **2004**, *6* (12), 963-966.

(4) Buono, P.; Duval, A.; Verge, P.; Averous, L.; Habibi, Y. New Insights on the Chemical Modification of Lignin: Acetylation versus Silylation. *ACS Sustain. Chem. Eng.* **2016**, *4* (10), 5212-5222.

(5) Zhu, G.; Zhao, F.; Wang, D.; Xia, C. Extended effective carbon number concept in the quantitative analysis of multi-ethers using predicted response factors. *J. Chromatogr. A* **2017**, *1513*, 194-200.

(6) Kim, H.; Ralph, J. A gel-state 2D-NMR method for plant cell wall profiling and analysis: a model study with the amorphous cellulose and xylan from ball-milled cotton linters. *RSC Adv.* **2014**, *4* (15), 7549-7560.
